# Synergetic Effect of Ultrasmall Metal Clusters and Zeolites Promoting Hydrogen Generation

**DOI:** 10.1002/advs.201802350

**Published:** 2019-03-25

**Authors:** Qiming Sun, Ning Wang, Risheng Bai, Yu Hui, Tianjun Zhang, David A. Do, Peng Zhang, Lijuan Song, Shu Miao, Jihong Yu

**Affiliations:** ^1^ State Key Laboratory of Inorganic Synthesis and Preparative Chemistry College of Chemistry Jilin University 2699 Qianjin Street Changchun 130012 P. R. China; ^2^ Key Laboratory of Petrochemical Catalytic Science and Technology Liaoning Province Liaoning Shihua University Fushun 113001 China; ^3^ Department of Chemistry Dalhousie University Halifax Nova Scotia B3H 4R2 Canada; ^4^ Dalian Institute of Chemical Physics Chinese Academy of Sciences Dalian 116023 P. R. China; ^5^ International Center of Future Science Jilin University 2699 Qianjin Street Changchun 130012 P. R. China

**Keywords:** ammonia borane, heterogeneous catalysis, hydrogen evolution, metal clusters, zeolites

## Abstract

Taking advantage of the synergetic effect of confined ultrasmall metal clusters and zeolite frameworks is an efficient strategy for improving the catalytic performance of metal nanocatalysts. Herein, it is demonstrated that the synergetic effect of ultrasmall ruthenium (Ru) clusters and intrinsic Brønsted acidity of zeolite frameworks can significantly promote the hydrogen generation of ammonia borane (AB) hydrolysis. Ultrasmall Ru clusters are embedded onto the silicoaluminophosphate SAPO‐34 (**CHA**) and various aluminosilicate zeolites (**MFI,** *BEA, and **FAU**) with tunable acidities by a facile incipient wetness impregnation method. Evidenced by high‐resolution scanning transmission electron microscopy, the sub‐nanometric Ru clusters are uniformly distributed throughout the zeolite crystals. The X‐ray absorption spectroscopy measurements reveal the existence of Ru‐H species between Ru clusters and adjacent Brønsted acid sites of zeolites, which could synergistically activate AB and water molecules, significantly enhancing the hydrogen evolution rate of AB hydrolysis. Notably, the Ru/SAPO‐34‐0.8Si (Si/Al = 0.8) and Ru/FAU (Si/Al = 30) catalysts with strong acidities afford high turnover frequency values up to 490 and 627 min^−1^, respectively. These values are more than a 13‐fold enhancement than that of the commercial Ru/C catalyst, and among the top level over other heterogeneous catalysts tested under similar conditions.

Hydrogen (H_2_) is one of the best environmentally friendly fuels and a promi‐sing efficient energy carrier for future applications because of its abundance, high energy density, and renewability.^1^ However, controllable and efficient storage and release of H_2_ remains a well‐known challenge for the establishment of the fuel‐cell‐based hydrogen economy.^2^ The ammonia borane (NH_3_BH_3_, AB) is considered to be one of the most fascinating candidates for chemical hydrogen storage for its higher hydrogen content, lower molecular weight, superior solubility in water, and stability at room temperature, as well as nontoxicity.^3^ In recent years, various supported metal catalysts have been developed for the hydrolysis of AB, among which ruthenium (Ru) has proven to be one of the most effective catalysts for H_2_ evolution.^4^ Several types of supports including metal oxides,^5^ carbon materials,^6^ g‐C_3_N_4_,^7^ metal–organic frameworks,^8^ and porous organic cages[qv: 4b,9] have been employed in AB dehydrogenation reactions. However, these catalysts usually possess larger particle sizes and nonuniform distributions of metal species and suffer from the relatively poor long‐term stability and unsatisfactory activity and recyclability. Therefore, developing highly efficient and stable catalysts for hydrolysis of AB is highly desired.

Recently, encapsulating metal species into zeolite matrices with well‐defined microporous structures has been demonstrated to be a powerful strategy for confinement synthesis of ultrasmall metal nanoparticles, sub‐nanometric clusters, and even single atoms.^10^ The zeolite nanospaces can efficiently inhibit the aggregation of the metal species, and thus improve the catalytic activity and stability of the nanocatalysts during the catalytic reactions. Significantly, zeolite frameworks can be endowed with acidity or basicity, which may integrate with the metal species, playing an important synergetic role in catalytic reactions. However, in most previous works, zeolites just act as an inert support, without utilizing their specific properties, such as the acidity and alkalinity.[qv: 10e–g] Stephens et al. reported that small amounts of strong acids, such as B(C_6_F_5_)_3_, HOSO_2_CF_3_, and HCl can initiate the dehydrocoupling of AB in organic solutions.^11^ Recently, Wang et al. found that the concentration of H^+^ ions in the reaction aqueous solution could greatly affect the catalytic activity of AB hydrolysis.[qv: 5b] It is expected that zeolites with intrinsic Brønsted acidity would be a superior support for confinement synthesis of ultrasmall metal clusters and further promise enhanced catalytic performance for AB hydrolysis taking advantage of the synergistic effect of zeolites and confined metal clusters. On the other hand, the nanosized zeolites, especially those with nanosheet‐like morphology are considered as ideal supports to anchor ultrasmall metal species with high dispersions due to their larger external surfaces compared with micron‐sized counterparts.[qv: 10h]

Herein, ultrasmall Ru clusters have been embedded onto nanosheet‐like silicoaluminophosphate SAPO‐34 zeolites (**CHA** zeotype) and various aluminosilicate zeolites (**MFI,** *BEA, and **FAU**) with tunable acidities via a facile incipient wetness impregnation method. The SAPO‐34 zeolite possesses a large *cha* cage (0.94 × 1.27 nm in diameter) and a 3D intersecting straight‐channel system of 8‐rings (0.38 nm), which is widely used in many important industrial processes, such as methanol‐to‐olefin and conversion of automobile exhaust.^12^ The schematic illustration of the structure of **CHA** is shown in Scheme S1 in the Supporting Information. Interestingly, we first find that the ultrasmall Ru clusters and adjacent Brønsted acid sites of SAPO‐34 zeolites can synergistically activate the AB and water molecules, and remarkably promote the H_2_ evolution rate from AB hydrolysis. Among all Ru/SAPO‐34 catalysts, the Ru/SAPO‐34‐0.8Si catalyst with the highest acid concentrations affords a high turnover frequency (TOF) value up to 490 min^−1^ at 25 °C. The catalytic performance of H_2_ generation can be further enhanced by using acidic aluminosilicate zeolites (**MFI**, *BEA, and **FAU**) as supports that possess stronger acidity and larger pore sizes than SAPO‐34 zeolites. Significantly, the Ru/FAU (dealuminated H‐type zeolite Y, Si/Al = 30) catalyst exhibits an extremely high TOF value reaching up to 627 min^−1^, which is the top level in the hydrolytic dehydrogenation of AB over heterogeneous catalysts under similar conditions.

As shown in **Figure** **1**A, ultrasmall Ru clusters supported on SAPO‐34 zeolites were prepared by impregnating suitable amounts of the RuCl_3_ solution in SAPO‐34 zeolites followed by H_2_ reduction. SAPO‐34 zeolites with different silicon contents were synthesized with the molar compositions of *x*SiO_2_: 1.0Al_2_O_3_: 1.2P_2_O_5_: 2.0 TEAOH: 33H_2_O (*x* = 0.1 and 0.2). The obtained samples were named as Ru/SAPO‐34‐xSi. As a comparison, the Ru/AlPO‐34 catalyst was also prepared by impregnating RuCl_3_ solution in AlPO‐34 zeolite synthesized with the similar initial gel of SAPO‐34 zeolites except without adding silicon species.

**Figure 1:**
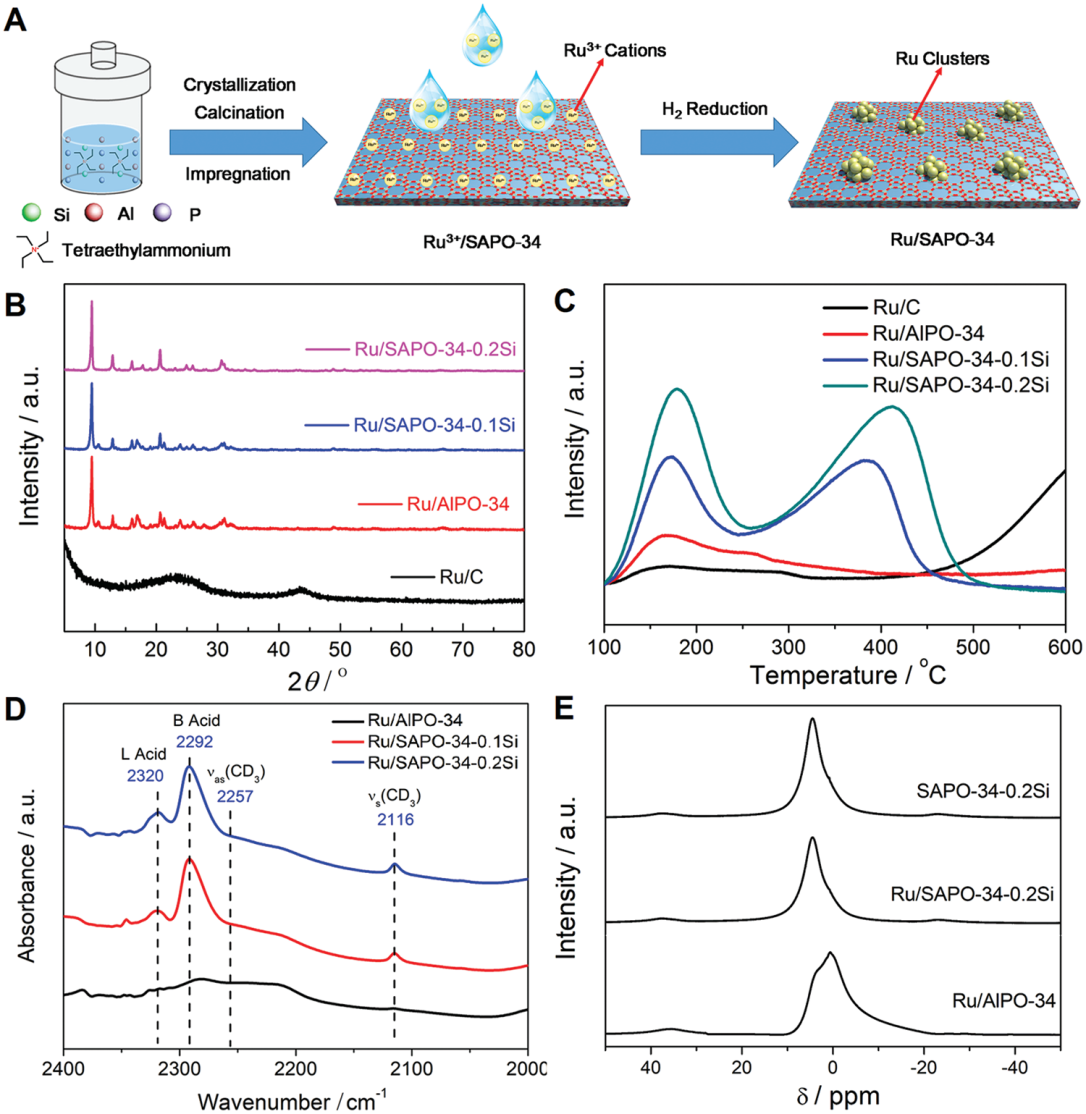
A) Synthetic procedure of Ru/SAPO‐34 catalysts, B) XRD patterns, C) NH_3_‐TPD curves, D) in situ IR spectroscopy of the adsorbed deuterated CD_3_CN, and E) ^1^H MAS NMR spectra of samples.

The X‐ray diffraction (XRD) patterns of the as‐prepared Ru/zeolite samples all display typical diffraction peaks of the **CHA** structure with high crystallinity (Figure 1B), indicating that introduction of Ru clusters does not break the zeolite^12^ The peaks of Ru metal cannot be detected in Ru‐containing zeolite samples due to the high dispersion and small particle size. Inductively coupled plasma (ICP) atomic emission spectroscopy analyses give that samples Ru/AlPO‐34, Ru/SAPO‐34‐0.1Si, and Ru/SAPO‐34‐0.2Si all possess a similar metal loading amount (0.43–0.44 wt%) (Table S1, Supporting Information). Compared with commercial Ru/C catalyst, ammonia temperature‐programmed desorption (NH_3_‐TPD) measurements reveal that the Ru/AlPO‐34 and Ru/SAPO‐34 catalysts possess acidic sites (Figure 1C). With the increase of the silicon content, the acidity of zeolite is further enhanced. Among all the samples, the Ru/SAPO‐34‐0.2Si sample possesses the highest acid strength and concentration due to the high silicon content in the framework. In situ infrared (IR) spectroscopy of the adsorbed deuterated acetonitrile (CD_3_CN) was used to probe the acidity of the catalysts. As shown in Figure 1D, two peaks at 2320 and 2292 cm^−1^, corresponding to stretchings of the ‐CN groups interacting with Lewis and Brønsted acid sites can be clearly observed in Ru/SAPO‐34‐0.1Si and Ru/SAPO‐34‐0.2Si, respectively, but these peaks are hardly visible in Ru/AlPO‐34. Among all obtained samples, Ru/SAPO‐34‐0.2Si possesses the highest acidic concentrations, and the numbers of total acid site, Brønsted acid site, and Lewis acid site are 0.57, 0.49, and 0.08 mmol g^−1^, respectively. The detailed acidic concentrations of various samples are summarized in Table S1 in the Supporting Information. The ^1^H MAS NMR spectra of samples Ru/AlPO‐34, Ru/SAPO‐34‐0.2Si, and SAPO‐34‐0.2Si are shown in Figure 1E. The signal at *≈*4.0 ppm can be clearly observed in both Ru/SAPO‐34‐0.2Si and pure SAPO‐34‐0.2Si samples, which are attributed to bridging OH groups (SiOHAl) with Brønsted acid sites.^13^ For the Ru/AlPO‐34 sample, the peak at *≈*1.0 ppm is the predominant signal corresponding to the TOH (T = P and Al) groups of framework defects.^13^

Transmission electron microscope (TEM) images of AlPO‐34 and SAPO‐34 zeolites are shown in Figure S1 in the Supporting Information, showing the nanosheet‐like morphology. Aberration‐corrected high angle annular dark field scanning TEM (HAADF‐STEM) images and Ru size distributions of Ru/AlPO‐34, Ru/SAPO‐34‐0.1Si, and Ru/SAPO‐34‐0.2Si samples are shown in **Figure** **2**A–F. The Ru clusters are well dispersed and uniformly distributed throughout the zeolite crystals. Some Ru clusters are located on the external surface of zeolites with the average Ru clusters sizes of about 1.5 nm, and some sub‐nanometric Ru clusters with several atoms are encapsulated inside the zeolite matrices, which are much smaller than that of the commercial Ru loaded over Ru/C catalyst (3.0 nm) (Figure S2, Supporting Information). The elemental mappings for Al, P, O, Si, and Ru elements of Ru/SAPO‐34‐0.2Si are shown in Figure 2G. These results reveal that the Ru and Si elements are distributed throughout the SAPO‐34 zeolite crystals. The energy‐dispersive X‐ray (EDX) spectrum further confirms the existence of Ru and Si in the sample (Figure S3, Supporting Information). N_2_ adsorption measurements show that about 0.03–0.05 cm^3^ g^−1^ of a decrease in micropore volume can be observed for Ru/zeolite samples as compared with the pure zeolite supports due to the partial occupation of the zeolite pores by the Ru clusters. However, these Ru‐containing zeolite samples still have sufficient void spaces (0.24–0.25 cm^3^ g^−1^) for the reactants and products transfer (Figures S4–S6 and Table S1, Supporting Information). In addition, the Ru/SAPO‐34‐0.2Si catalyst exhibits almost the same ^29^Si, ^31^P, and ^27^Al MAS NMR spectra as pure SAPO‐34‐0.2Si zeolite (Figures S7–S9, Supporting Information), indicating that the zeolite framework keeps intact after the metal clusters are supported on the zeolites.

**Figure 2:**
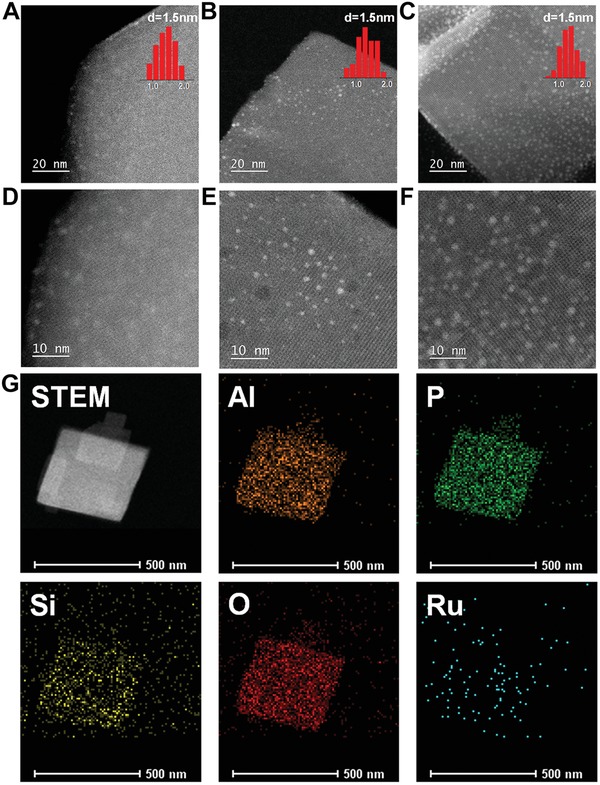
HAADF‐STEM images of A,D) Ru/AlPO‐34, B,E) Ru/SAPO‐34‐0.1Si and C,F) Ru/SAPO‐34‐0.2Si samples. G) HAADF‐STEM images of Ru/SAPO‐34‐0.2Si sample, and corresponding element maps showing distributions of Al, P, Si, O, and Ru, respectively.

X‐ray absorption near edge structure (XANES) and extended X‐ray absorption fine structure (EXAFS) of Ru/zeolite samples are shown in **Figure** **3**A–C. The Ru K‐edge XANES spectra of Ru/zeolite samples show some differences from Ru foil due to the small particle sizes of Ru clusters and the interaction between Ru clusters and the zeolite framework. Interestingly, with the increase of zeolite acidity, the main peaks at the Ru K‐edge XANES spectra at around 22.14 keV are gradually widened, decreasing in maximum intensity and shifting to higher energy. Concurrently, the absorption features at about 22.163 keV are increased along with the increase of zeolite acidity. All these changes in the XANES have been proven both experimentally and theoretically as the signature of hydrogen adsorption on transition metals.^14^ In our system, these shifts of peaks could be attributed to the formation of Ru‐H species between Ru clusters and adjacent Brønsted acid sites of SAPO‐34 zeolites, and the interaction of Ru‐H species are increased gradually with the increase of zeolite acidity. The formation of Ru‐H species could be explained that the Ru ions prefer to be absorbed near the acidic sites of zeolites due to the electric attraction during the impregnation process. Note that the coordination numbers (CNs) of Ru‐O shells from EXAFS fittings are reduced along with the increase of Brønsted acid sites of SAPO‐34 zeolite. This might be related to the existence of Ru‐H species, as H‐adsorption on the Ru cluster surface would naturally reduce the number of surface Ru—O bonds (Table S2, Supporting Information). Notably, the average CNs of the Ru—Ru metallic bonds in all of Ru/zeolite samples are only 1.8–2.1, indicating that the Ru clusters possess ultrasmall sizes and the average amount of Ru atoms of Ru clusters should be less than four (tetrahedral configuration, CN = 3). To further determine the valence of Ru clusters, the X‐ray photoelectron spectroscopy (XPS) analyses of the samples were also performed. As shown in Figure 3D, very weak XPS signals can be detected in Ru/SAPO‐34‐0.2Si due to the surface‐anchored Ru clusters. When the Ru/SAPO‐34‐0.2Si sample is dissolved in NaOH solution, two peaks at 462.5 and 484.8 eV corresponding to the Ru 3p_3/2_ and Ru 3p_1/2_ of Ru (0) can be clearly observed in the residual sample, and those peaks are similar to those of commercial Ru/C catalyst.^15^

**Figure 3:**
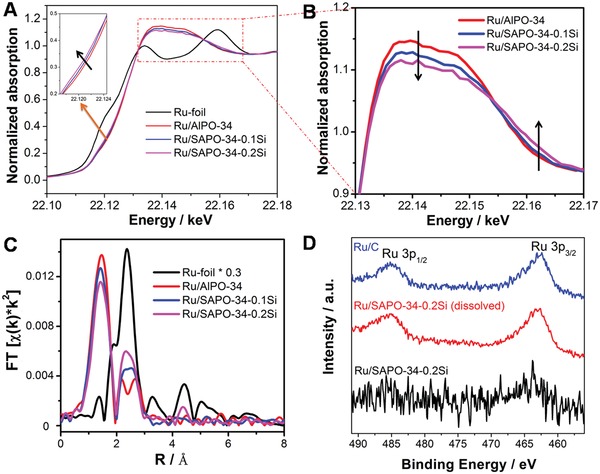
A,B) Ru K‐edge XANES spectra of Ru foil, Ru/zeolite samples. C) Fourier transform of k^2^‐weighted EXAFS spectra of catalysts at Ru K‐edge. D) Ru 3p XPS spectra of the samples.

Catalytic activities for H_2_ evolution from the dehydrogenation of AB (1 m) at 25 °C catalyzed over different catalysts are presented in **Figure** **4**A. It can be clearly seen that the Ru/SAPO‐34 catalysts are more active for the hydrolysis of AB than the commercial Ru/C catalyst due to the significantly reduced Ru clusters sizes. Notably, the H_2_ generation rates over the Ru/SAPO‐34 catalysts are much faster than that over the Ru/AlPO‐34 catalyst. Considering the similar Ru cluster sizes and contents of Ru/SAPO‐34 and Ru/AlPO‐34 catalysts, the improvement of catalytic performance over Ru/SAPO‐34 catalysts should be attributed to the existence of Brønsted acid sites of SAPO‐34 zeolite. Moreover, with the increase of acidity, the H_2_ evolution rate is getting faster. Significantly, the Ru/SAPO‐34‐0.2Si catalysts with the highest acid strength and concentration among these catalysts exhibit the best catalytic performance. 73.5 mL of H_2_, corresponding to H_2_/AB = 3, can be generated within 1.33 min (*n*_Ru_/*n*_AB_ = 0.007) toward complete decomposition of AB, affording high TOF value of 310 min^−1^ at 25 °C. This TOF value is of about sevenfold enhancement than that of the commercial Ru/C catalyst (46 min^−1^). Based on the gas chromatography analysis, the H_2_ is the only gas product from AB hydrolysis (Figure S10, Supporting Information). ^1^H NMR and ^11^B NMR spectra before and after reactions indicate that the NH_4_^+^ and BO_2_^−^ species are the final products besides H_2_ (Figures S11 and S12, Supporting Information), which is consistent with previous works.[qv: 3c,16] According to the aforementioned catalytic results, the decomposition of AB can be formulated as below:

$${\mathrm{NH}}_{3}{\mathrm{BH}}_{3}+2H_{2}O\overset{\mathrm{Ru/SAPO‐34}}{\to }{\mathrm{NH}}_{4}^{+}+{\mathrm{BO}}_{2}^{-}+3H_{2}↑$$

**Figure 4:**
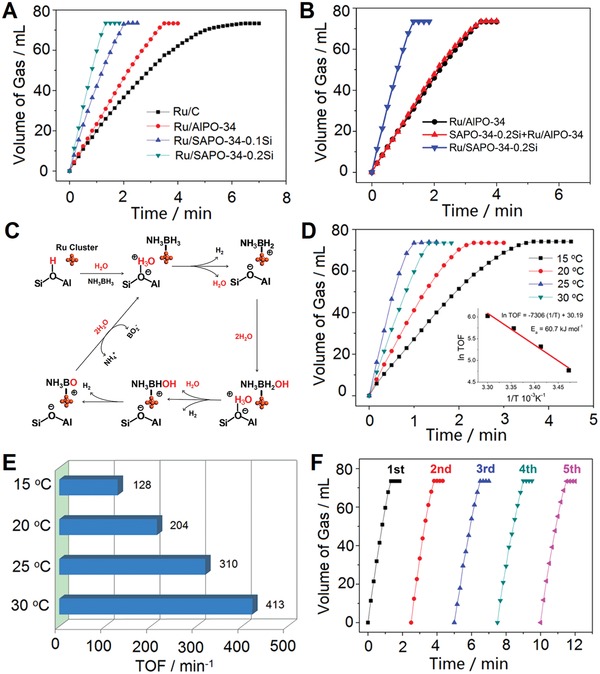
A,B) Volume of the H_2_ generated from AB (1 m) hydrolysis versus time at 25 °C catalyzed by various catalysts (*n*_Ru_/*n*_AB_ = 0.007). C) The proposed mechanism for NH_3_BH_3_ hydrolysis over Ru/SAPO‐34 catalysts. D) Volume of the H_2_ generated from AB (1 m) hydrolysis versus time and E) corresponding TOF values at different temperatures catalyzed by Ru/SAPO‐34‐0.2Si catalyst (*n*_Ru_/*n*_AB_ = 0.007), inset of (D): Arrhenius plot (ln TOF versus 1/T). F) Durability tests for the AB (1 m) hydrolysis at 25 °C over Ru/SAPO‐34‐0.2Si catalysts (*n*_Ru_/*n*_AB_ = 0.007).

To investigate the effect of the acidity on the AB decomposition, firstly, the catalytic reactions of AB hydrolysis over commercial Ru/C catalyst were performed at various pH values (pH = 5–8) conditions by using different buffer solutions replacing the aqueous solution. The control experiments of AB hydrolysis at the same pH values without catalysts were also measured. In the absence of catalysts, with the decrease of pH values, the volume of generated H_2_ was increased, but the total H_2_ volumes were less than 3.4 mL (about 5% of AB decomposition) within 10 min (Figure S13, Supporting Information). After adding commercial Ru/C catalyst, the catalytic H_2_ generation rates were clearly fast and improved along with the decrease of pH values (Figure S14, Supporting Information). These results reveal that the increased H^+^ ion concentration in reaction solutions could enhance the decomposition of AB, especially with introducing metallic catalysts. Next, we performed the catalytic reactions of AB hydrolysis over the pure AlPO‐34 and SAPO‐34‐0.2Si zeolites. At 25 °C, less than 0.4 mL of H_2_ could be detected over these zeolite samples. When the reaction temperature increased up to 50 °C, about 4.8 mL H_2_ could be generated from pure SAPO‐34‐0.2Si zeolite within 10 min, which was fourfold improvement than that of pure AlPO‐34 zeolite (1.2 mL) (Figures S15 and S16, Supporting Information), confirming that the Brønsted acid sites of SAPO‐34 zeolite are beneficial for the decomposition of AB. Furthermore, the physical mixture of Ru/AlPO‐34 and SAPO‐34‐0.2Si catalysts exhibited no improvement for the H_2_ generation rate as compared with the pure Ru/AlPO‐34 catalyst (Figure 4B), indicating that the synergistic effect between Ru clusters and adjacent Brønsted acid sites of zeolite supports is responsible for enhancement of the catalytic activity for the H_2_ evolution. According to all above catalytic results, the possible mechanism for NH_3_BH_3_ hydrolysis over Ru/SAPO‐34 catalysts is proposed in Figure 4C. On the one hand, the Ru clusters can adsorb the AB molecules and activate the cleavage of B—H bonds[qv: 5b,11]; on the other hand, the adjacent Brønsted acid sites of zeolites can simultaneously activate the water and the proton transfer can proceed from acidic zeolites to water inside the zeolite framework.^17^ The protonated water molecules are prone to bond with the dissociated H atoms from B—H bonds in the AB molecules to form the H_2_ molecules. The Ru clusters and adjacent Brønsted acid sites of zeolite could act as bi‐functional active sites to synergistically activate the ammonia borane and water molecules, respectively, thus significantly promoting the H_2_ generation from the AB hydrolysis. Moreover, the nanosheet‐like morphology of SAPO‐34 zeolites is also a key point to enhance the catalytic activity of H_2_ generation. To illustrate the issue, a control Ru/SAPO‐34‐0.2Si‐TEA catalyst (Ru nanoparticles located onto the micron‐sized SAPO‐34‐0.2Si‐TEA crystals) was synthesized by using triethylamine as the template. As shown in Figures S17 and S18 in the Supporting Information, the Ru/SAPO‐34‐0.2Si‐TEA catalyst possesses a micron‐scale size (*≈*10 µm) of zeolites, and most of Ru nanoparticles are located onto the outer surface of the Ru/SAPO‐34‐0.2Si‐TEA crystals. The Ru/SAPO‐34‐0.2Si‐TEA has the similar metal loading amount (0.44 wt%) but larger particle size of Ru (*≈*3 nm) than that of the Ru/SAPO‐34‐0.2Si catalyst synthesized by using TEAOH as the template. The NH_3_‐TPD measurements reveal that the Ru/SAPO‐34‐0.2Si‐TEA possesses similar acidic strength and concentration compared with the Ru/SAPO‐34‐0.2Si sample (Figure S19, Supporting Information). However, the hydrogen generation rate of AB hydrolysis catalyzed by Ru/SAPO‐34‐0.2Si‐TEA catalysts (TOF = 200 min^−1^) are much lower than that of Ru/SAPO‐34‐0.2Si sample (TOF = 310 min^−1^) (Figure S20, Supporting Information).

Figure 4D,E show the H_2_ evolution from the AB hydrolysis at different reaction temperatures over Ru/SAPO‐34‐0.2Si catalyst. With the increase of reaction temperature, the H_2_ evolution rates are improved. The apparent activation energy of Ru/SAPO‐34‐0.2Si catalyst is 60.7 kJ mol^−1^, which is comparable with those reported in previous works for AB hydrolysis.^18^ The durability tests for the AB hydrolysis over Ru/SAPO‐34‐0.2Si catalyst were also investigated. After the previous run, the Ru/SAPO‐34‐0.2Si catalyst was washed with water. The dried catalyst was reused for the catalytic dehydrogenation of AB. We found that with the increase of recycling numbers, the rate of H_2_ generation over Ru/SAPO‐34‐0.2Si catalyst was decreased gradually, but still higher than that of Ru/AlPO‐34 catalyst (Figure S21, Supporting Information). The NH_3_‐TPD analysis shows that the acidity of Ru/SAPO‐34‐0.2Si catalyst after three cycles decreases sharply compared to that of the fresh catalyst (Figure S22, Supporting Information). This might be due to that some intermediates such as NH_3_BO^+^ or NH_4_^+^ species (see Figure 4C) cover onto the acidic sites of zeolites. To recovery the acidic sites of zeolite, after the previous run, the Ru/SAPO‐34‐0.2Si catalyst was extra mixed with a formic acid solution (0.1 m), stirred for 15 min, and then washed to neutral with water. The NH_3_‐TPD analysis shows that the Ru/SAPO‐34‐0.2Si catalyst treated with the acid could recover most of the acidic sites as compared with the fresh catalyst (Figure S22, Supporting Information). After five cycles, the activity of H_2_ generation of the acid‐treated catalyst was much the same as that of the fresh catalyst (Figure 4F). Significantly, after the catalytic reactions, the size distributions of Ru clusters, as well as the morphology and the metal content of the Ru/SAPO‐34‐0.2Si catalyst keep unchanged (Figure S23, Supporting Information). This demonstrates that the Ru/SAPO‐34 catalyst possesses excellent recycling stabilities during AB hydrolysis, and further confirms that the acidity of zeolites plays an important role in the improvement of the catalytic activity of AB hydrolysis.

To further enhance the catalytic activity of AB hydrolysis, the Ru/SAPO‐34‐0.4Si (SiO_2_/Al_2_O_3_ = 0.4), Ru/SAPO‐34‐0.6Si (SiO_2_/Al_2_O_3_ = 0.6), and Ru/SAPO‐34‐0.8Si (SiO_2_/Al_2_O_3_ = 0.8) catalysts with the similar metal loading amounts (*≈*0.43 wt%) and higher silicon contents compared with those of Ru/SAPO‐34‐0.2Si were also synthesized. TEM images of these catalysts show that the average particle sizes of Ru clusters are about 1.5 nm, which are similar to that of the Ru/SAPO‐34‐0.2Si catalyst (Figure S24, Supporting Information). NH_3_‐TPD measurements reveal that the acidity of SAPO‐34 zeolites improves gradually in line with the increase of silicon contents. Among them, the Ru/SAPO‐34‐0.8Si exhibits the highest acidic concentration and strength (**Figure** **5**A). Based on the in situ IR spectroscopy of the adsorbed CD_3_CN measurement, the total and Brønsted acid sites of Ru/SAPO‐34‐0.8Si reach up to 0.76 and 0.67 mmol g^−1^, respectively (Figure S25, Supporting Information). Notably, the H_2_ generation rate catalyzed by Ru/SAPO‐34‐0.8Si (TOF = 490 min^−1^) is much faster than that of Ru/SAPO‐34‐0.2Si (TOF = 310 min^−1^) at 25 °C (Figure 5B). This further indicates that the increased acidity may promote the proton transfer proceeding from acidic zeolites to water inside the zeolite framework, enhance the synergetic catalytic effect between Ru clusters and Brønsted acid sites of SAPO‐34 zeolites, and thus improve the catalytic performance of AB hydrolysis.

**Figure 5:**
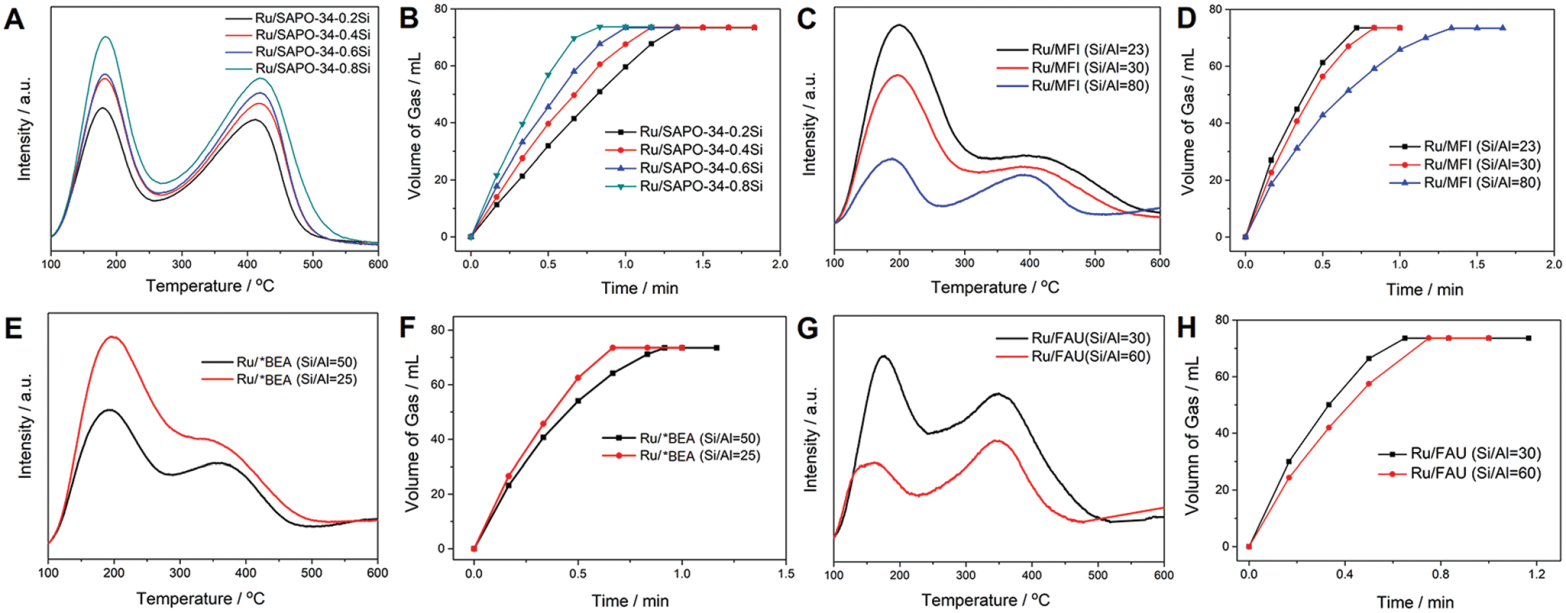
NH_3_‐TPD curves of A) Ru/SAPO‐34 catalysts, C) Ru/MFI catalysts, E) Ru/*BEA catalysts, and G) Ru/FAU catalysts. Volume of the H_2_ generated from AB (1 m) hydrolysis versus time at 25 °C catalyzed by B) Ru/SAPO‐34 catalysts (TOF values of Ru/SAPO‐34‐0.2, Ru/SAPO‐34‐0.4, Ru/SAPO‐34‐0.6, and Ru/SAPO‐34‐0.8 are 310, 356, 415, 490 min^−1^, respectively), D) Ru/MFI catalysts (TOF values of Ru/MFI (Si/Al = 80), Ru/MFI (Si/Al = 30), and Ru/MFI (Si/Al = 23) are 302, 497, and 575 min^−1^, respectively), F) Ru/*BEA catalysts (TOF values of Ru/*BEA (Si/Al = 50) and Ru/*BEA (Si/Al = 25) are 501 and 615 min^−1^, respectively), and H) Ru/FAU catalysts (TOF values of Ru/*BEA (Si/Al = 60) and Ru/*BEA (Si/Al = 30) are 522 and 627 min^−1^, respectively). The ratios of *n*_Ru_/*n*_AB_ are all fixed for 0.007.

Based on the above experimental results, it can be expected that other zeolites with stronger acidity than SAPO‐34 (e.g., aluminosilicate zeolites) may act as promising supports for further enhancing the catalytic performance of an H_2_ generation. To verify our suppose and further elucidate the effect of zeolite acidity on H_2_ production, the Ru clusters (*≈*0.43 wt%) were also supported onto a series of commercial H‐type acidic aluminosilicate zeolites with different Si/Al ratios, such as **MFI**, *BEA, and **FAU** (dealuminated zeolite Y) zeolites (Figures S26–S28, Supporting Information). TEM images show that the Ru clusters are uniformly distributed throughout all aluminosilicate zeolite supports and the average particle sizes of Ru clusters are less than 2 nm, which is quite similar to that of Ru/SAPO‐34 catalysts (Figures S29–S31, Supporting Information). As expected, the H_2_ generation rates are promoted along with the increase of zeolite acidities, indicating the general concept that the synergetic effect of Ru clusters and Brønsted acid sites of zeolites can enhance the catalytic performance of AB hydrolysis (Figure 5C–H). Significantly, the TOF values of Ru/FAU (Si/Al = 30), Ru/*BEA (Si/Al = 25), and Ru/MFI (Si/Al = 23) reach up to 627, 615, and 575 min^−1^, respectively, which are higher than those of their corresponding counterparts with lower acidities and that of the Ru/SAPO‐34‐0.8Si (TOF = 490 min^−1^), and are more than 13‐fold enhancement than that of the commercial Ru/C catalyst. These TOF values are much higher than that of the best Ru‐based heterogeneous catalysts for AB decomposition reported so far under similar conditions,[qv: 4b,19] and other metal‐based heterogeneous catalysts (Table S3, Supporting Information).[qv: 5b,9,20] Compared with Ru/SAPO‐34 catalysts, the enhanced catalytic performance of these catalysts can be attributed to the increased acidic strength that can further improve the synergetic effect of Ru clusters and acidic sites of zeolites for AB hydrolysis. As shown in Figures S32–S34 in the Supporting Information, the vibration peaks in IR spectra between adsorbed CD_3_CN and acid sites are still observable on the Ru/FAU (Si/Al = 30) and Ru/*BEA (Si/Al = 25), when the desorption temperature rises to 200 °C. However, these peaks almost disappear in the Ru/SAPO‐34‐0.8Si at 200 °C, indicating that Ru/FAU (Si/Al = 30) and Ru/*BEA (Si/Al = 25) possess stronger acidity than Ru/SAPO‐34‐0.8Si. On the other hand, the enlarged pore size of these aluminosilicate zeolites (10‐ or 12‐ring) can also enhance the transport efficiency of AB molecules, which is beneficial for the interaction between AB molecules and Ru clusters confined with zeolite frameworks and improving the catalytic performance of AB hydrolysis.

In summary, ultrasmall Ru clusters have been successfully anchored onto SAPO‐34 and various alunimosilica zeolites (**MFI**, *BEA, and **FAU**) with tunable acidities by a facile incipient wetness impregnation method. The ultrasmall Ru clusters and adjacent Brønsted acid sites of zeolite could act as bi‐functional active sites to synergistically activate the ammonia borane and water molecules, which can significantly promote the H_2_ generation from the AB hydrolysis. Meanwhile, the catalytic activity of AB hydrolysis can be improved with the increase of zeolite acidities. Notably, thanks to the synergetic effect between ultrasmall Ru clusters and high acid strength and concentration of zeolites, the Ru/SAPO‐34‐0.8Si and Ru/FAU (dealuminated H‐type zeolite Y, Si/Al = 30) catalysts afford extremely high TOF values toward the complete decomposition of AB, reaching up to 490 and 627 min^−1^ at 25 °C, respectively. These values are much higher than that of the best Ru‐based heterogeneous catalysts and are the top level among all metal‐based heterogeneous catalysts for AB decomposition reported so far under similar conditions. The work provides a useful guidance for the design of high‐efficient nanocatalysts by taking advantage of the synergetic effect of zeolites and metal clusters. Moreover, the excellent catalytic activity and simple synthetic method of zeolite‐supported metal nanocatalysts promise their practical application of chemical hydrogen storage in a fuel cell‐based hydrogen economy in future.

## Conflict of Interest

The authors declare no conflict of interest.

## Supporting information

Supplementary
