# Supplementary material for: Synergetic Effect of Ultrasmall Metal Clusters and Zeolites Promoting Hydrogen Generation
Source: Adv Sci (Weinh). 2019 Mar 25;6(10):1802350. doi: 10.1002/advs.201802350 (PMC6524121; doi:10.1002/advs.201802350)
Supplement: Supplementary file 1 — Supplementary [file ADVS-6-1802350-s001.pdf]

Copyright WILEY-VCH Verlag GmbH & Co. KGaA, 69469 Weinheim, Germany, 2019.

ADVANCED SCIENCE
Open Access

Supporting Information

for *Adv. Sci.,* DOI: 10.1002/advs.201802350

Synergetic Effect of Ultrasmall Metal Clusters and Zeolites Promoting Hydrogen Generation

*Qiming Sun, Ning Wang, Risheng Bai, Yu Hui, Tianjun Zhang, David A. Do, Peng Zhang, Lijuan Song, Shu Miao, and Jihong Yu**

Copyright WILEY-VCH Verlag GmbH & Co. KGaA, 69469 Weinheim, Germany,

2019.

Supporting Information

Synergetic Effect of Ultrasmall Metal Clusters and Zeolites Promoting Hydrogen Generation

Qiming Sun, Ning Wang, Risheng Bai, Yu, Hui, Tianjun Zhang, David A. Do, Peng Zhang, Lijuan Song, Shu Miao, Jihong Yu*

Dr. Q. Sun, Dr. N. Wang, Mr. R. Bai, Mr. T. Zhang, Prof. J. Yu
State Key Laboratory of Inorganic Synthesis and Preparative Chemistry, College of Chemistry, Jilin University, 2699 Qianjin Street, Changchun 130012, People's Republic of China.
E-mail: jihong@jlu.edu.cn

Mr. Y. Hui, Prof. L. Song
Key Laboratory of Petrochemical Catalytic Science and Technology, Liaoning Province, Liaoning Shihua University, Fushun 113001, China

Dr. D, Do, Prof. P. Zhang
Department of Chemistry, Dalhousie University, Halifax, Nova Scotia B3H 4R2, Canada.

Prof. S. Miao
Dalian Institute of Chemical Physics, Chinese Academy of Sciences, Dalian 116023, People's Republic of China.

Prof. J. Yu
International Center of Future Science, Jilin University, 2699 Qianjin Street, Changchun 130012, People's Republic of China

1 Experimental Sections and Characterizations

Chemicals and Materials. Tetraethylammonium hydroxide solution (TEAOH, 35 wt%, Alfa Aesar), aluminium iso-propoxide ((Al(O^{i}Pr)$_3$, Beijing Reagents Company), phosphoric acid (H_3PO_4, 85 wt%, Beijing Chemical Works), colloidal silica (40 wt%, Aldrich), ruthenium(III) chloride anhydrous ($RuCl_3$, Aladdin), deionized water from Millipore (Milli-Q, 18.2 MΩ/cm; Millipore, Bedford, MA), ammonia borane (NH_3BH_3, AB, 90 wt%, Aladdin), Ru/C (5 wt%, Aladdin), acetic acid (CH_3COOH, HAc, Xilong Scientific Co., Ltd.), sodium acetate (CH_3COONa, NaAc, Beijing Reagents Company), sodium dihydrogen phosphate (NaH_2PO_4, Beijing Reagents Company), disodium phosphate dodecahydrate ($Na_2HPO_4·12H_2O$, Beijing Reagents Company), triethylamine (TEA, 99%, Fuyu Company). The commercial acidic **MFI**, ***BEA**, and **FAU** (dealuminated H-type zeolite Y) zeolites with different Si/Al ratios was purchased in the Alfa Aesar chamicals company. For comparability, the Si/Al ratios of all aluminosilicate zeolites are in the range of 25 to 80.

Synthesis of nanosheet-like SAPO-34 and AlPO-34 zeolites. The nanosheet-like SAPO-34 zeolites were synthesized under conventional hydrothermal conditions at 170 °C for 3 days from the starting gel with the molar compositions of 1.0 Al_2O_3: 1.2 P_2O_5: x SiO_2: 2.0 TEAOH: 33 H_2O (x = 0.1, 0.2, 0.4, 0.6, and 0.8), the obtained samples were named as SAPO-34-0.1Si, SAPO-34-0.2Si, SAPO-34-0.4Si, SAPO-34-0.6Si, and SAPO-34-0.8Si, respectively.

Typically, 10.2 g of finely ground Al(O^{i}Pr)$_3$ powder was mixed with 21 g of TEAOH solution and suitable amount of water, followed by a continuous stirring for 2 h. Then, 6.92 g of phosphoric acid was dropwise added into the above mixture, followed by a drastically stirring for 2 h. Finally, the suitable amount of colloidal silica was slowly added. The reaction mixture was stirred for 1 h and then transferred into a 100 mL Teflon-lined stainless steel autoclave. The crystallization was conducted in a conventional oven at 170 °C for 3 days under static conditions. The as-synthesized solid products were centrifuged, washed with water and ethanol for several times, and then dried at 80 °C in the oven overnight, followed by calcination at 550 °C for 6 h.

The nanosheet-like $AlPO_4$-34 zeolite was prepared from the starting gel with the molar composition of 1.0 Al_2O_3: 1.2 P_2O_5: 2.0 TEAOH: 33 H_2O under the same condition for the synthesis of SAPO-34 zeolites except without adding the colloidal silica.

Synthesis of Ru/SAPO-34 and Ru/AlPO-34 catalysts. Ru/SAPO-34-xSi and Ru/AlPO-34 catalysts were prepared by the incipient wetness impregnation method. Typically, 1 g of calcined SAPO-34 or AlPO-34 zeolite was impregnated with $RuCl_3$ solution (0.23 mL, 0.19 M), and then the mixture was drastically stirred to allow the $RuCl_3$ solution absorbed into the zeolites. The obtained solid was dried at 80 °C in the oven overnight, and then reduced sequentially in flowing H_2 with linear heating to 400 °C for 2 h and holding for 2 h.

Synthesis of micron-sized SAPO-34-0.2Si-TEA and Ru/SAPO-34-0.2Si-TEA. The micron-sized SAPO-34-0.2Si-TEA zeolite was synthesized with a molar compositions of 1.0 Al_2O_3: 1.0 P_2O_5: 4.7 TEA: 0.2 SiO_2: 70H_2O by using the triethylamine as the template under hydrothermal conditions at 200 ℃ for 36 h. The Ru/SAPO-34-0.2Si-TEA was prepared by the same incipient wetness impregnation method with the Ru/SAPO-34 catalysts.

Characterizations. The crystallinity and phase purity of the samples were characterized by powder X-ray diffraction (PXRD) on a Rigaku D/Max 2550 diffractometer using Cu Kα radiation (λ = 1.5418 Å). The transmission electron microscopy (TEM) images and corresponding energy dispersive X-ray (EDX) spectrometry were measured with a Tecnai F20 electron microscope. Cs-corrected HAADF-STEM images were taken on a JEM-ARM200F scanning transmission electron microscope (STEM) operated at 200 kV. The metal contents of all samples were determined with inductively coupled plasma (ICP) analyses on a Perkin-Elmer Optima 3300 DV ICP instrument. Nitrogen adsorption/desorption measurements were carried out on a Micromeritics 2020 analyzer at 77.35 K after the samples were degassed at 350 °C under vacuum. The temperature-programmed desorption of ammonia (NH_3-TPD) experiments were performed using a Micromeritics Auto Chem II 2920 automated chemisorption analysis unit equipped with a thermal conductivity detector (TCD) under helium flow. The ^{29}Si, ^{27}Al, ^{31}P, and ^{1}H MAS NMR measurements were performed on Bruker AVANCE III 400 WB spectrometer at a magnetic field strength of 9.4 T. The resonance frequencies were 79.5, 104.2, 161.9, and 400.1 MHz for ^{29}Si,

^{27}Al, ^{31}P, and ^{1}H, respectively. The spinning rate of all samples at the magic angle was 12 kHz. The chemical shifts were referenced to 85% H_3PO_4 solution for ^{31}P, 1 M $Al(NO_3)_3$ solution for ^{27}Al, 2,2-dimethyl-2-ilapentane-5-sulfonate sodium salt (DSS) for ^{29}Si and ^{1}H, respectively. the X-ray photoelectron spectroscopy (XPS) was measured by ESCALAB 250 spectrometer. To obtain the valence of Ru clusters in the catalysts, the dissolved samples were prepared. The brief steps are listed as follows: the Ru/SAPO-34-0.2Si catalyst was first dissolved in suitable amount of NaOH solution (10 M). After stirring, the residual solid was then isolated from the mixture by centrifugation, washed with water, and dried with vacuum freeze dryer. The released gas was analyzed using Agilent GC 6890N, equipped with thermal conductivity detector (TCD) and Plot-Q column (Agilent J&W GC Columns, HP-PLOT/Q 19095P-Q04, 30m × 530μm × 40μm). Liquid NMR spectra were recorded on a BRUKER AVANCEIII 500 MHz spectrometer (500.13 MHz for ^{1}H NMR and 160.42 MHz for ^{11}B NMR). Liquid samples of the filtrates, in which D_2O was included as solvent or a lock, were contained in sample tubes of 5 mm.

The X-ray absorption spectroscopy data were collected at the Sector 20-BM beamline of the Advanced Photon Source at Argonne National Laboratory. Sample powders were packed on Kapton tapes and folded multiple times to enhance the signal. The beamline was equipped with a double-crystal Si (111) monochromator. A 12-element Ge fluorescence detector was used to collect spectra of the Ru K-edge. Data processing and EXAFS fitting were performed using the WinXAS software in conjunction with scattering path amplitude and phase functions calculated using the FEFF8 program.

The FTIR spectra were scanned between 4000 and 1200 cm^{-1} after the adsorbed samples degassed at temperatures of 30, 100, 200, 300 °C for 1 h, respectively, using a Perkin-Elmer Spectrum TM GX spectrometer. Samples were first pressed into self-supporting discs with a diameter of 15 mm. Subsquently, the samples were pre-treated in the IR cell attached to a vacuum line at 100 °C (1.2 °C /min) for 1.5 h, and then at 450 °C (2 °C /min) for 2 h under 10^{-6} Torr. The adsorption of the deuterated acetonitrile was performed at 30 °C. After establishing a pressure of 10 torr at equilibrium, in order to remove the physisorbed species, the cell was evacuated at 30 °C. The concentrations of Brønsted and Lewis acid sites were determined from the quantitative analyses of the characteristic IR bands at 2292 and 2320 cm^{-1}, respectively. The molar extinction

coefficient obtained for Lewis acid sites was 3.6 cm·μmol^{-1} and for Brønsted acid sites was 2.0 cm·μmol^{-1}.

2 Catalytic Tests

Hydrolysis Reaction of Ammonia Borane. The hydrolysis of AB reactions were carried out with an apparatus containing a reaction unit and a gas collecting device. In general, 0.5 mL distilled water was first placed in a two-necked round-bottomed flask (25 mL), and then suitable amounts of catalysts (for example 0.167 g of Ru/SAPO-34-0.2Si) was added in the flask (the molar ratio of Ru/AB of all catalysts were fixed at 0.007). The flask was placed in a water bath with magnetic stirring (600 rpm) at a preset temperature (15~30 °C) under ambient atmosphere. The volume of released gas was measured using a gas burette, and an electronic balance, continuously recording the weight of excurrent water. After the water level became balanced, the reaction was immediately started when 0.5 mL of the mixed aqueous AB solution (2.0 M) was injected into the flask using a syringe.

To investigate the influence of the H^+ ion, hydrolytic dehydrogenations of ammonia borane were carried out at 298K, using buffer solutions with different pH value (pH = 5.0~8.0) instead of water. The details of buffer solutions listed as follow: (a) pH=5.0 (0.15 mL HAc solution (0.4 M) and 0.35 mL NaAc solution (0.4M)); (b) pH=6.0 (0.439 mL of NaH_2PO_4 solution (0.4M) and 0.061 mL of Na_2HPO_4 solution (0.4M)); (c) pH=7.0 (0.19 mL of NaH_2PO_4 solution (0.4M) and 0.31 mL of Na_2HPO_4 solution (0.4M)); (d) pH=8.0 (0.026 mL of NaH_2PO_4 (0.4M) solution and 0.474 mL of Na_2HPO_4 solution (0.4M)).

To investigate the effect for Brønsted acid site of zeolites, the control experiments of hydrolysis for AB reactions were performed over the pure AlPO-34, and SAPO-34-0.2Si zeolites at 25 °C and 50 °C. The weights of the pure zeolites for the AB hydrolysis were the same as their corresponding Ru-containing zeolite catalysts.

Durability Test of Catalysts for Ammonia Borane Hydrolysis. After the completion of previous run, the catalyst was isolated from the reaction solution by centrifugation. The isolated catalyst was added into 0.1 M formic acid solution, following a continuous stirring for 15 min to remove some alkaline species on the zeolites, and then the isolated catalysts were washed with

water until the supernatant became neutral. The dried catalysts were reused in the catalytic dehydrogenation of AB solution. The reacted catalysts were reused up to 5 consecutive catalytic cycles. The Ru clusters sizes and distributions of isolated catalysts were characterized by transmission electron microscopy.

The Calculation of Turnover Frequency (TOF). The total turnover frequency (TOF) was calculated based on the quantity of Ru metal atoms in the catalysts when the conversion of AB reached up to 100%. The calculation equation used was as below:

$$TOF = \frac{PV_{H_2}/RT}{n_{Ru}t}$$

Where P is the atmospheric pressure (101.325 kPa), V_{H_2} is the total volume of the released gas, R is the universal gas constant (8.3145 m^3 Pa mol^{-1} K^{-1}), T is the room temperature (298 K), n_{Ru} is the mole of Ru atoms in catalyst, and the t is the completion time of the reaction in minute.

3 Supplementary Figures and Tables

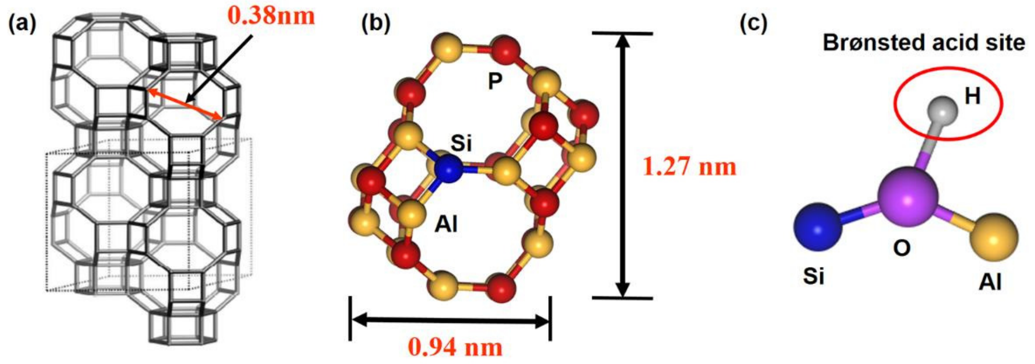

Scheme S1. Schematic illustration of (a) **CHA** structure, (b) cha cage of SAPO-34 zeolite, and (c) Brønsted acid site of SAPO-34 zeolite. To show the structure of CHA (SAPO-34) zeolite more clearly, only the T atoms (P, Al, and Si) were demonstrated in (a) and (b), and the oxygen atoms were omitted.

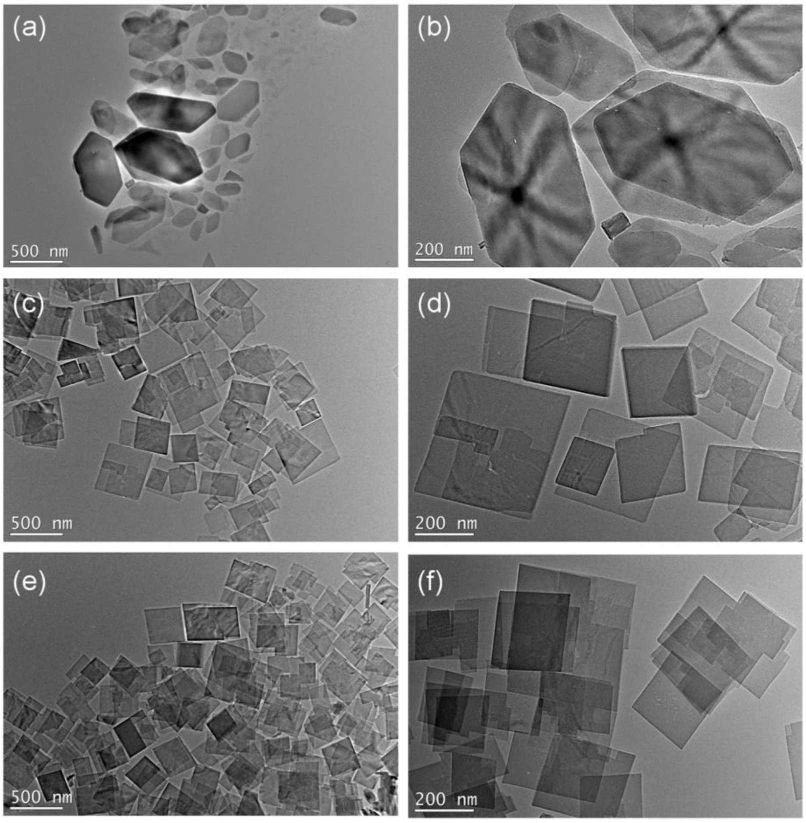

Figure S1. TEM images of AlPO-34 (a, b), SAPO-34-0.1Si (c, d) and SAPO-34-0.2Si (e, f).

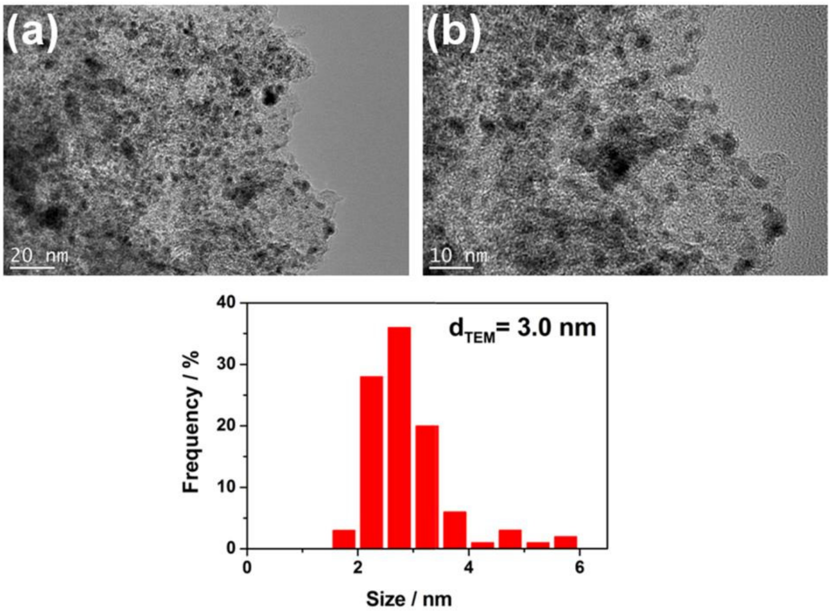

Figure S2. TEM images of Ru/C catalyst and corresponding size distributions of Ru clusters.

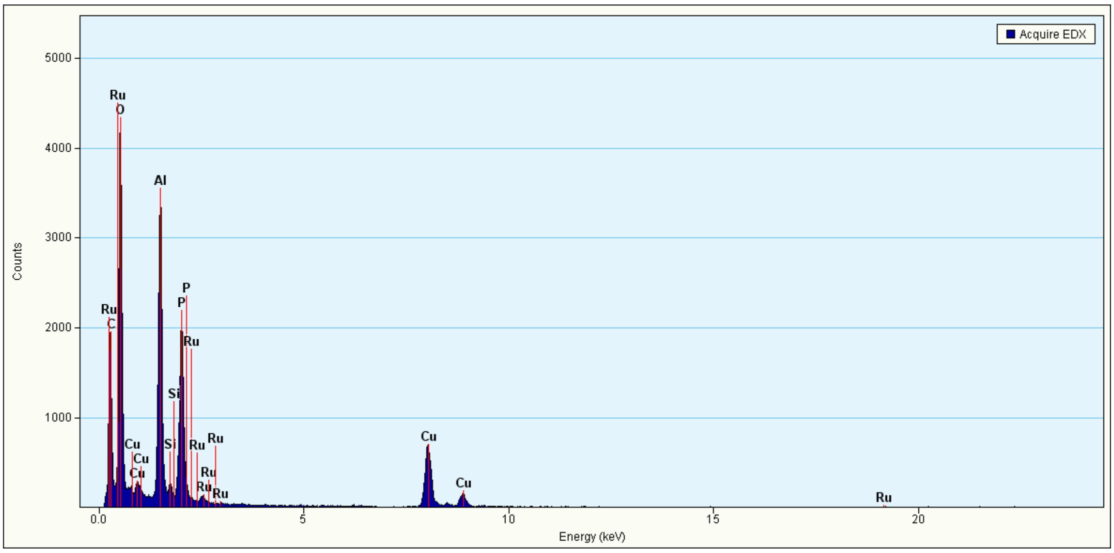

Figure S3. EDX spectrum of Ru/SAPO-34-0.2Si catalyst.

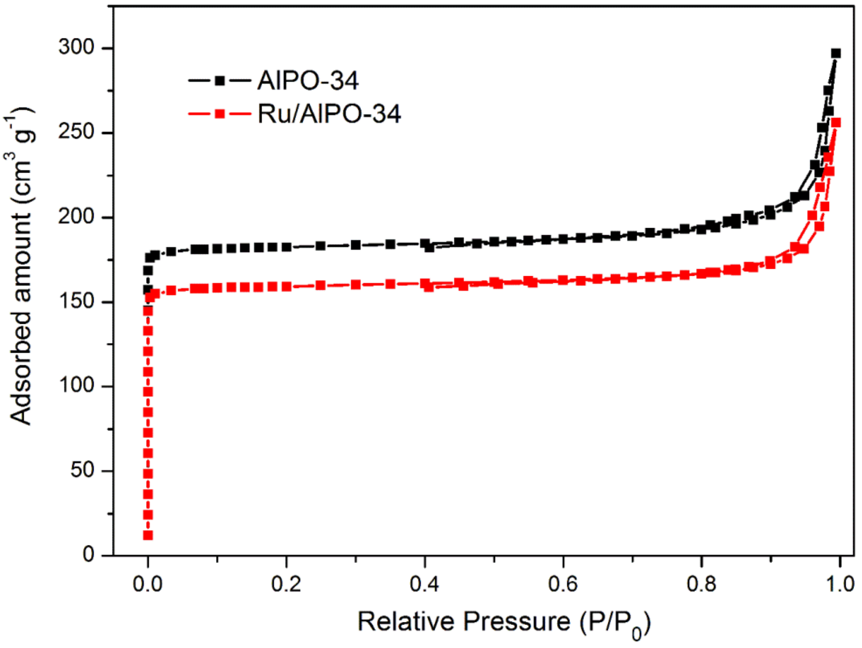

Figure S4. N_2 adsorption/desorption isotherms of samples AlPO-34 and Ru/AlPO-34.

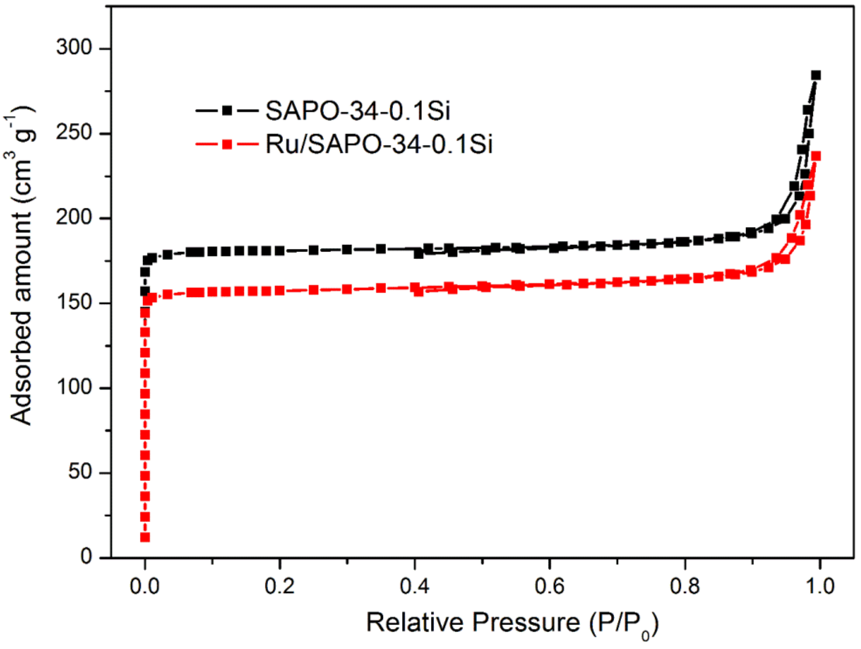

Figure S5. N_2 adsorption/desorption isotherms of samples SAPO-34-0.1Si and Ru/SAPO-34-0.1Si.

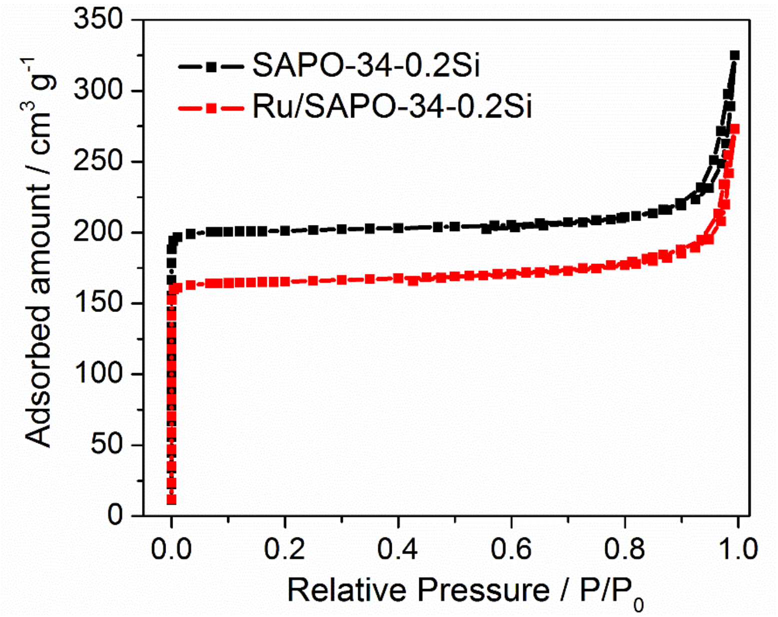

Figure S6. N_2 adsorption/desorption isotherms of samples SAPO-34-0.2Si and Ru/SAPO-34-0.2Si.

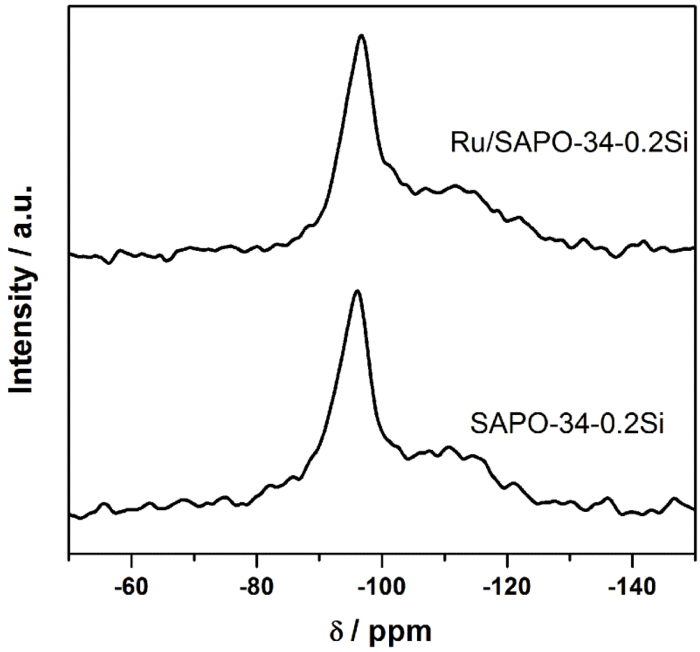

Figure S7. ^{29}Si MAS NMR spectra of samples SAPO-34-0.2Si and Ru/SAPO-34-0.2Si.

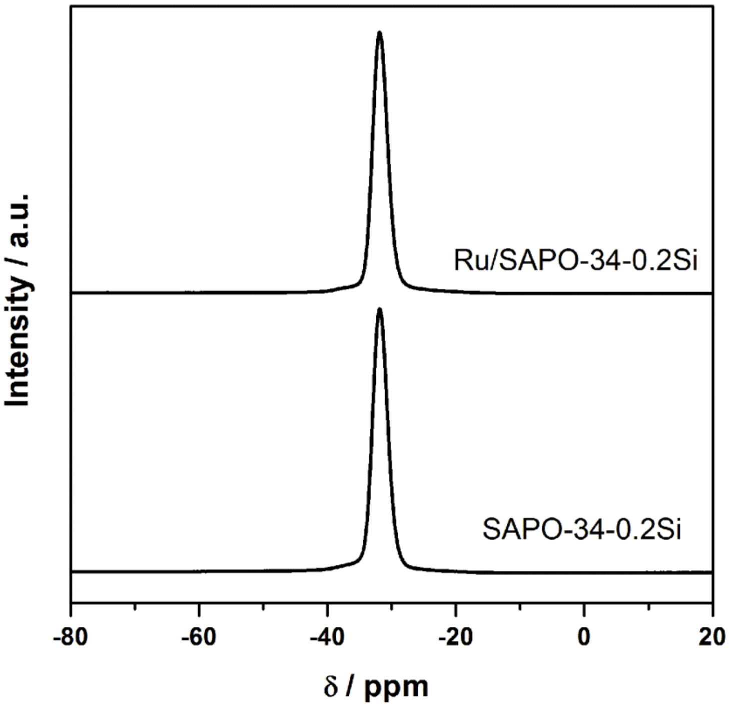

Figure S8. ^{31}P MAS NMR spectra of samples SAPO-34-0.2Si and Ru/SAPO-34-0.2Si.

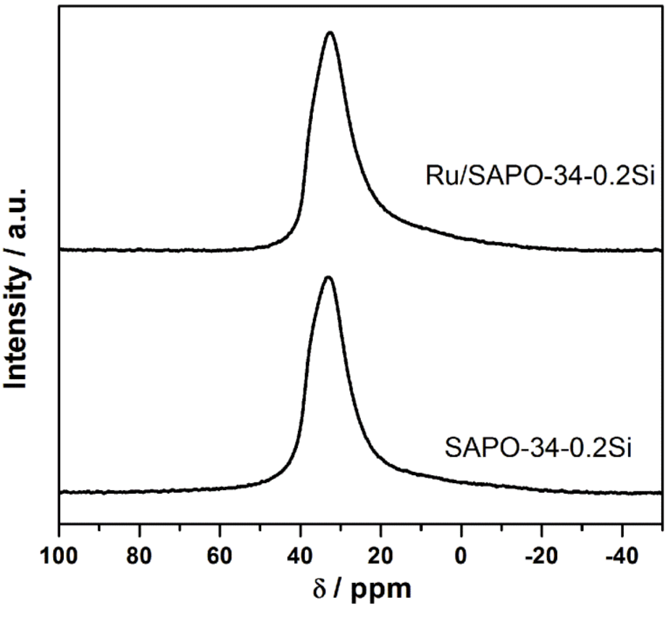

Figure S9. ^{27}Al MAS NMR spectra of samples SAPO-34-0.2Si and Ru/SAPO-34-0.2Si.

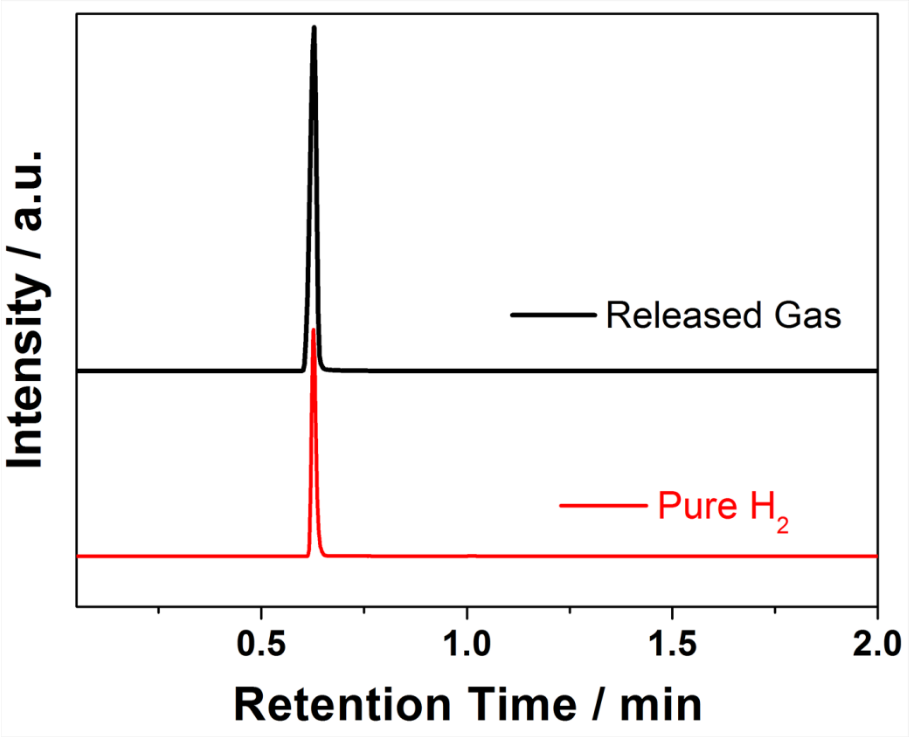

Figure S10. GC spectra using Agilent GC 6890N equipped with TCD detector for the evolved gas from AB hydrolysis over Ru/SAPO-34-0.2Si catalyst at 25 °C (n_{Ru}/n_{AB} = 0.007) as compared with pure H_2.

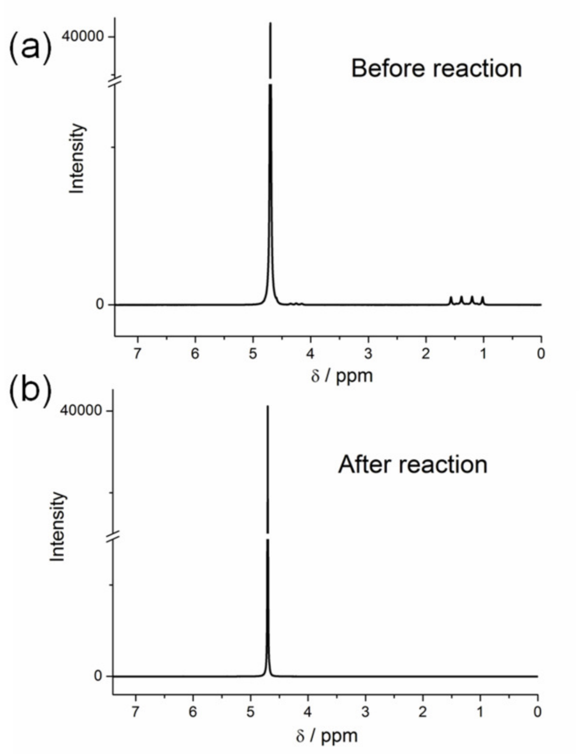

Figure S11. ^{1}H NMR spectra of the NH_3BH_3 solution in D_2O (a) before and (b) after reactions.

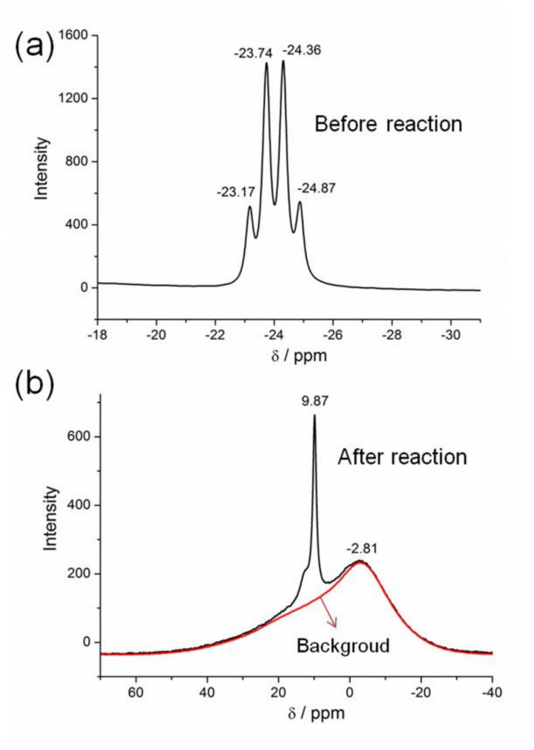

Figure S12. ^{11}B NMR spectra of the NH_3BH_3 solution in D_2O (a) before and (b) after reactions.

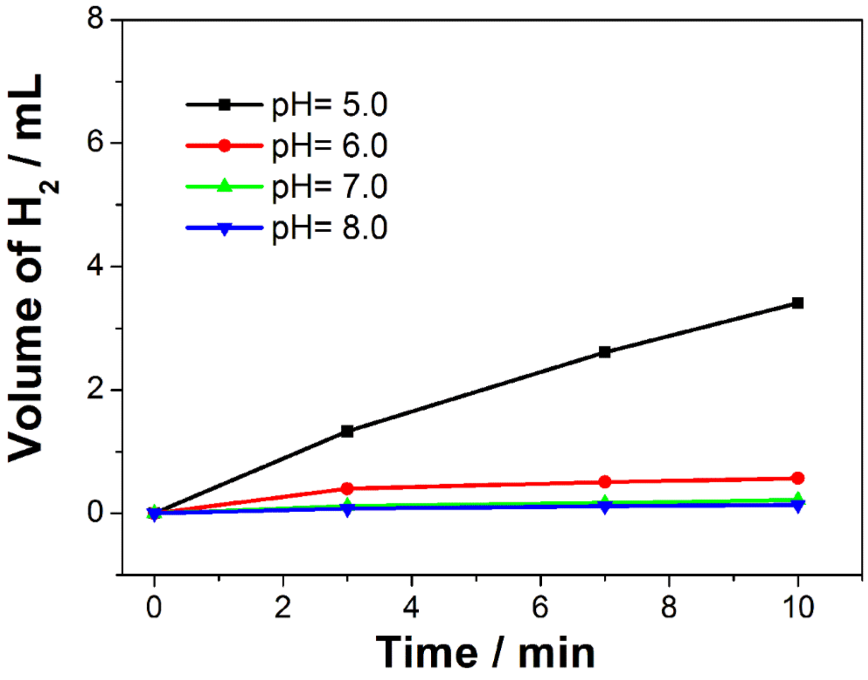

Figure S13. Volume of the H_2 generated from AB (1 M) hydrolysis at 25 °C with different pH values without adding catalysts.

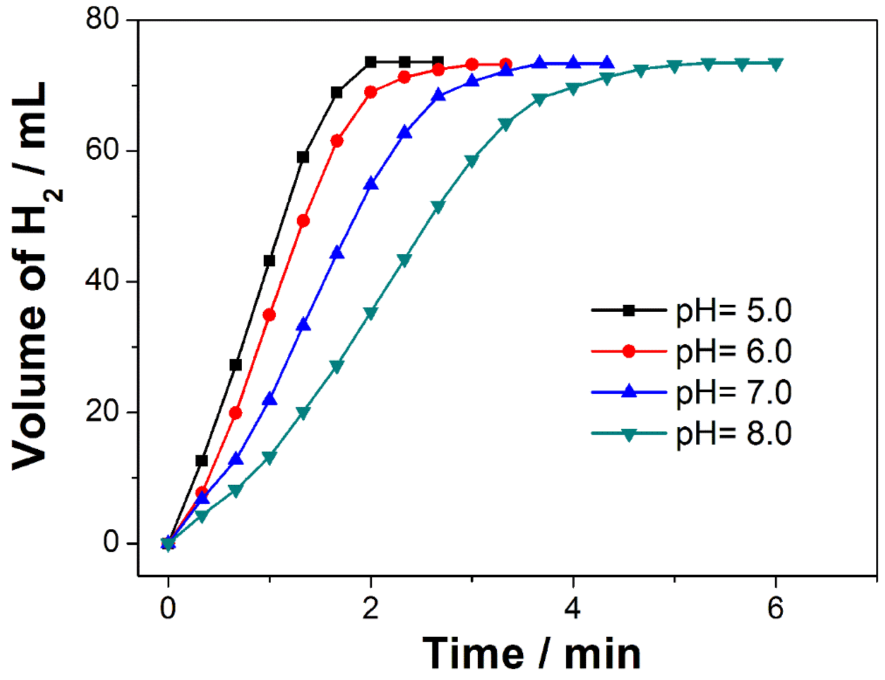

Figure S14. Volume of the H_2 generated from AB (1 M) hydrolysis at 25 °C with different pH values over Ru/C catalysts.

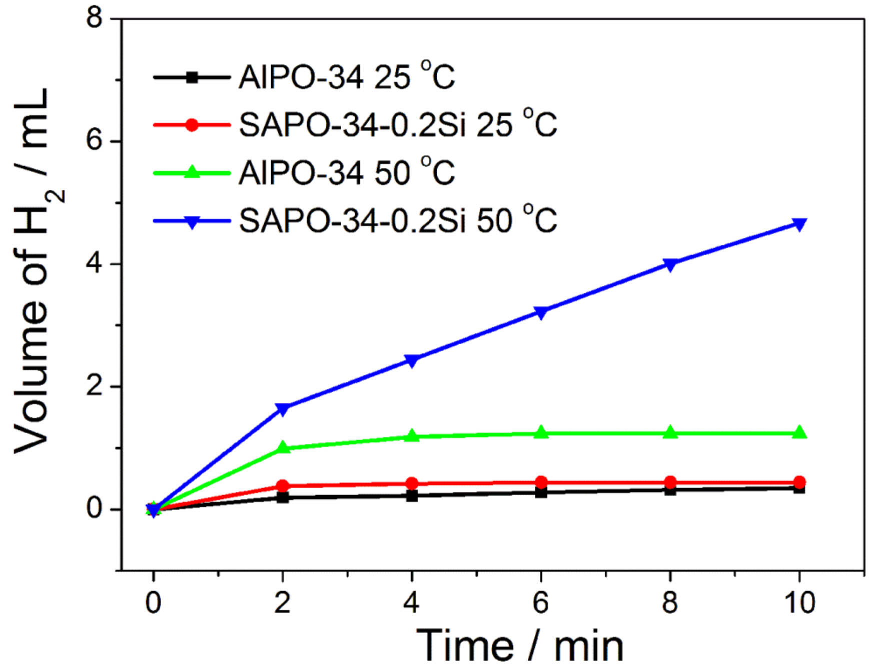

Figure S15. Volume of the H_2 generated from AB (1 M) hydrolysis over AlPO-34 and SAPO-34 catalysts at 25 °C and 50 °C.

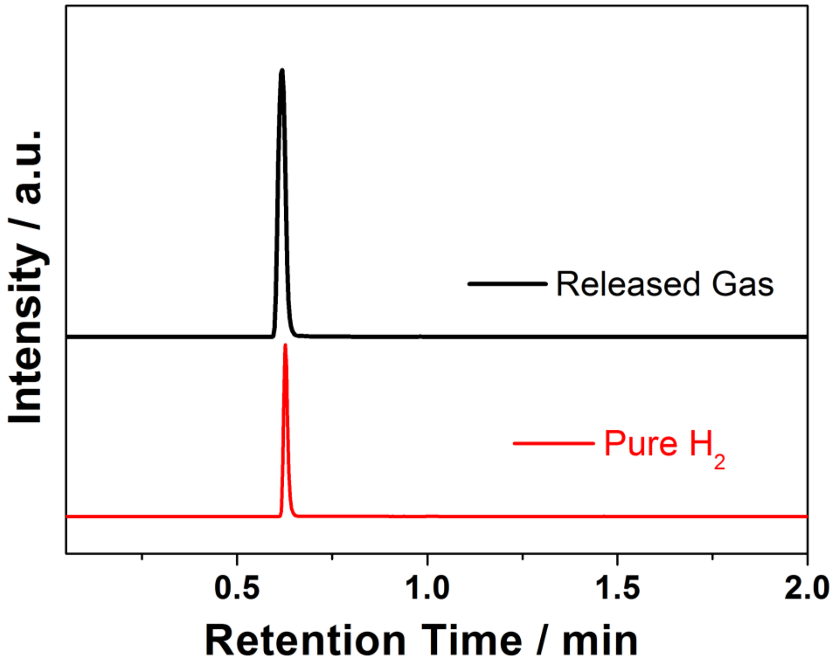

Figure S16. GC spectra using Agilent GC 6890N equipped with TCD detector for the evolved gas from AB hydrolysis over SAPO-34-0.2Si catalyst at 50 °C as compared with pure H_2.

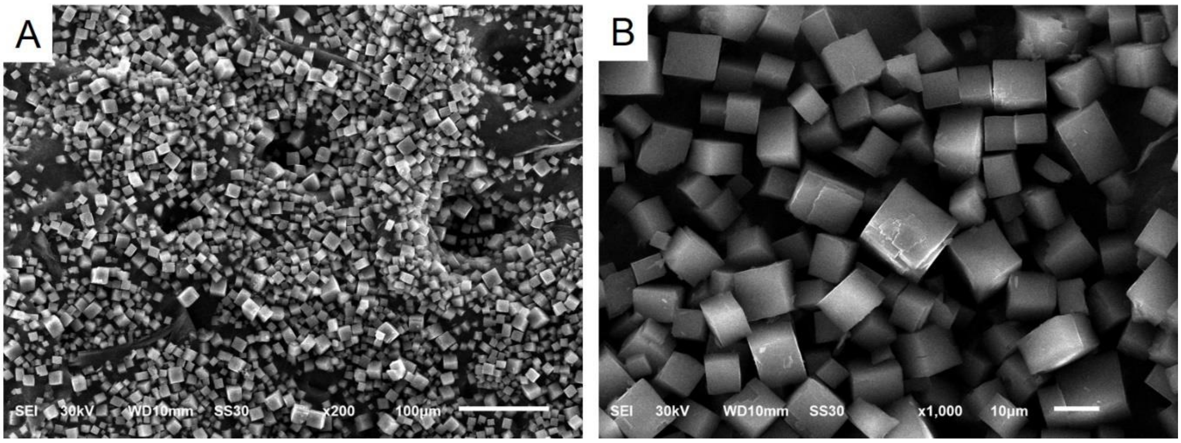

Figure S17. SEM images of Ru/SAPO-34-0.2Si-TEA samples.

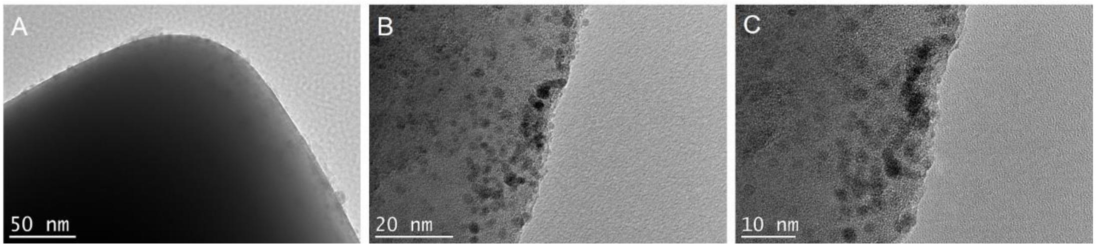

Figure S18. TEM images of Ru/SAPO-34-0.2Si-TEA samples.

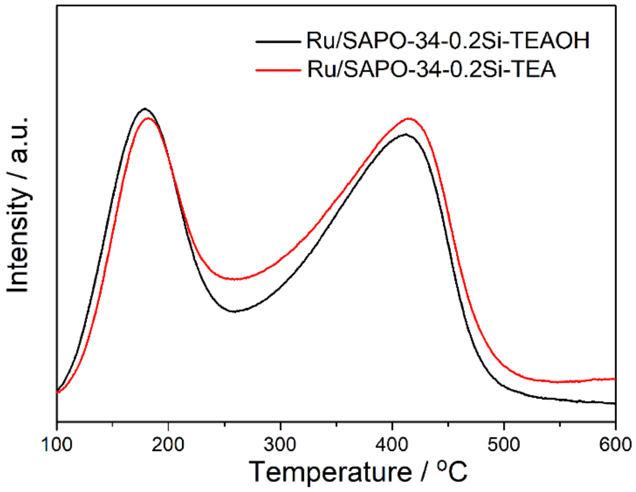

Figure S19. NH_3-TPD curves of the Ru/SAPO-34-0.2Si-TEA and Ru/SAPO-34-0.2Si-TEAOH samples.

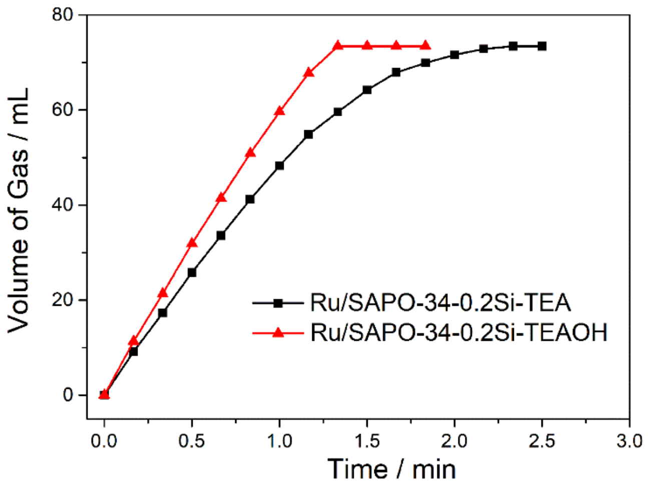

Figure S20. Volume of the H_2 generated from AB (1 M) hydrolysis versus time at 25 ^{o}C catalyzed by Ru/SAPO-34-0.2Si-TEA and Ru/SAPO-34-0.2Si-TEAOH catalysts (n_{Ru}/n_{AB} = 0.007).

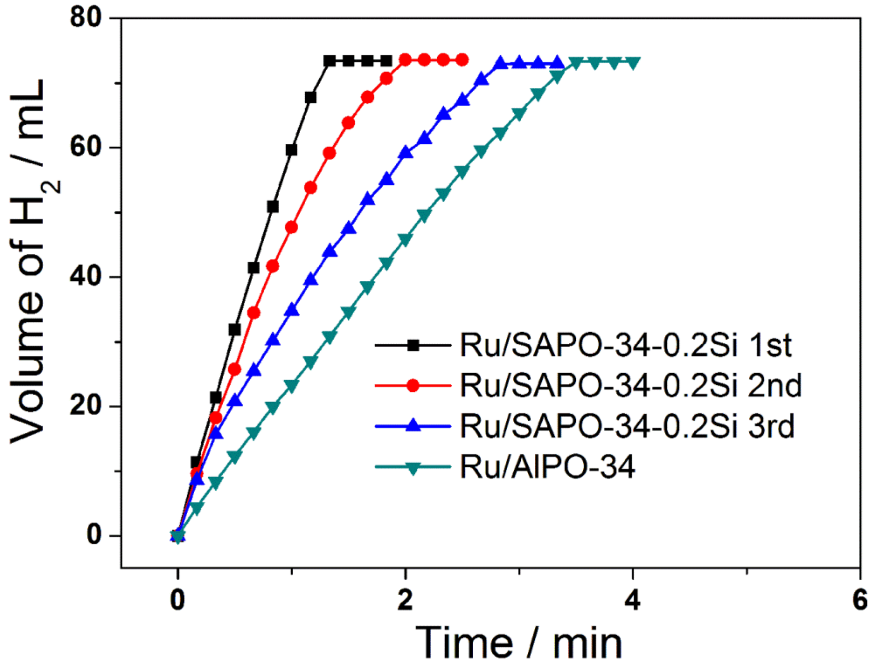

Figure S21. Durability tests for the AB (1 M) hydrolysis at 25 °C over Ru/SAPO-34-0.2Si catalyst (n_{Ru}/n_{AB} = 0.007) as compared with the Ru/AlPO-34 catalyst. After previous run, the Ru/SAPO-34-0.2Si catalyst was washed with water.

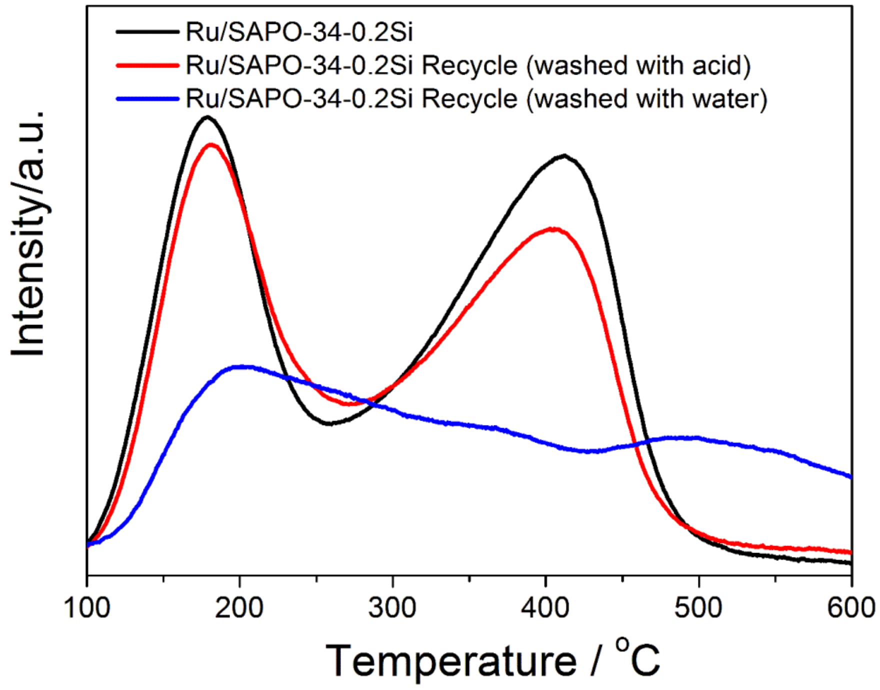

Figure S22. NH_3-TPD curves of fresh Ru/SAPO-34-0.2Si catalyst, and recycled catalysts Ru/SAPO-34-0.2Si (washed with acid) and Ru/SAPO-34-0.2Si (washed with water).

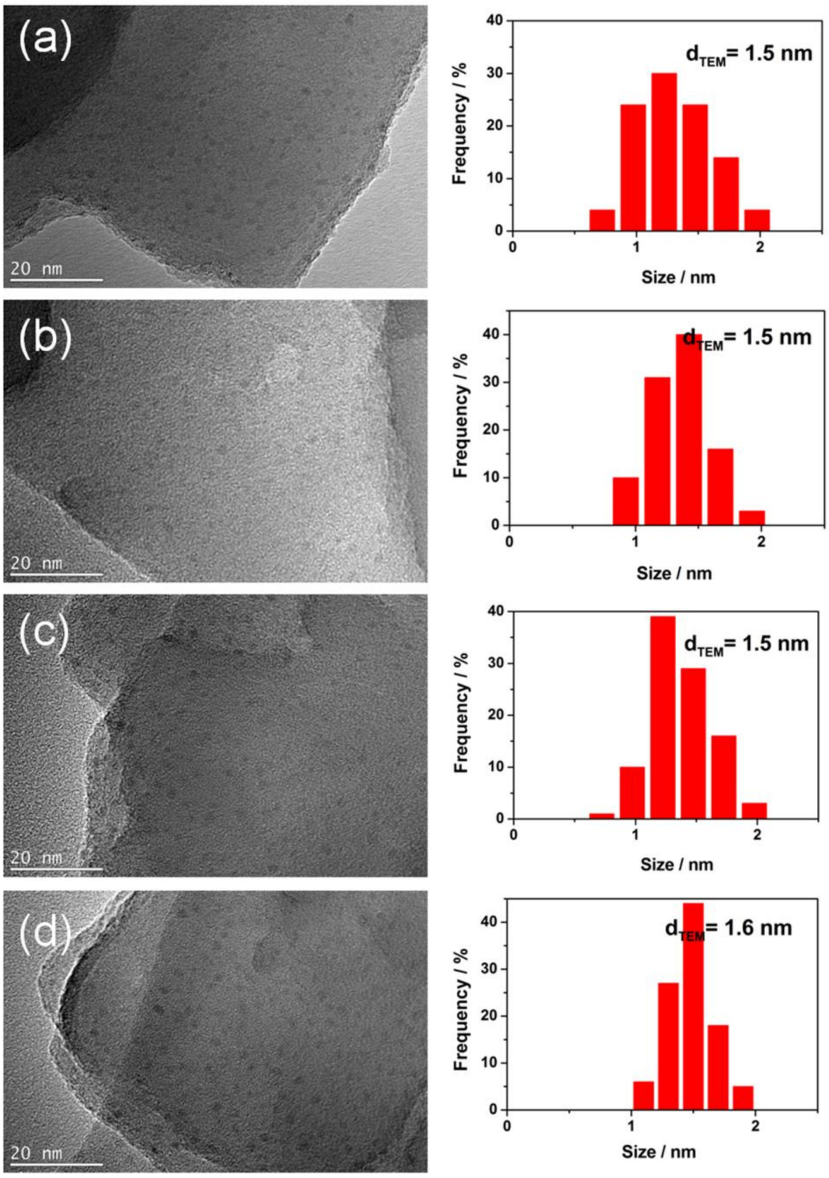

Figure S23. TEM images and corresponding size distributions of Ru clusters of Ru/SAPO-34-0.2Si catalysts (washed with acid) after durability tests of AB hydrolysis. (a) Fresh sample, (b) 1st recycle, (c) 3rd recycle and (d) 5th recycle.

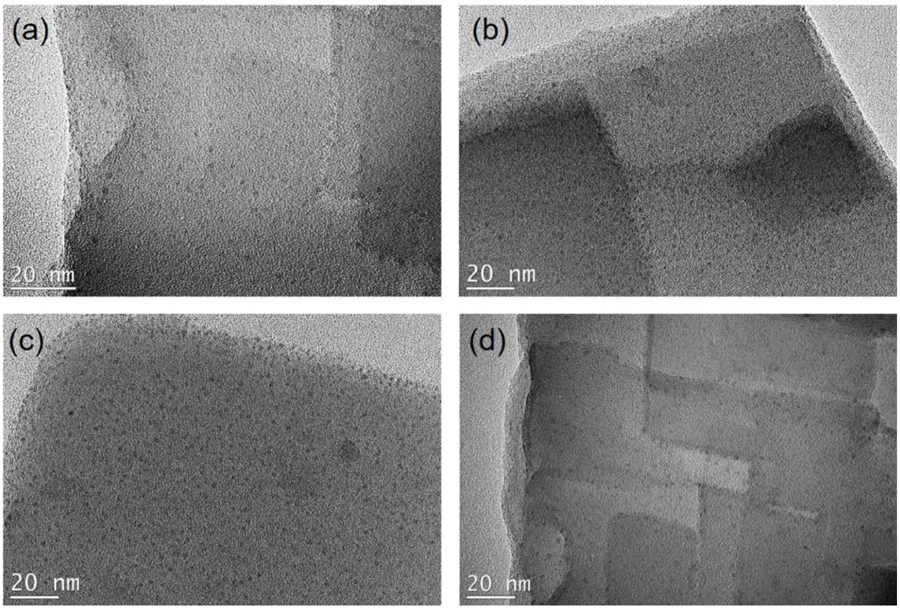

Figure S24. TEM images of (a) Ru/SAPO-34-0.2Si, (b) Ru/SAPO-34-0.4Si, (c) Ru/SAPO-34-0.6Si, and (d) Ru/SAPO-34-0.8Si catalysts.

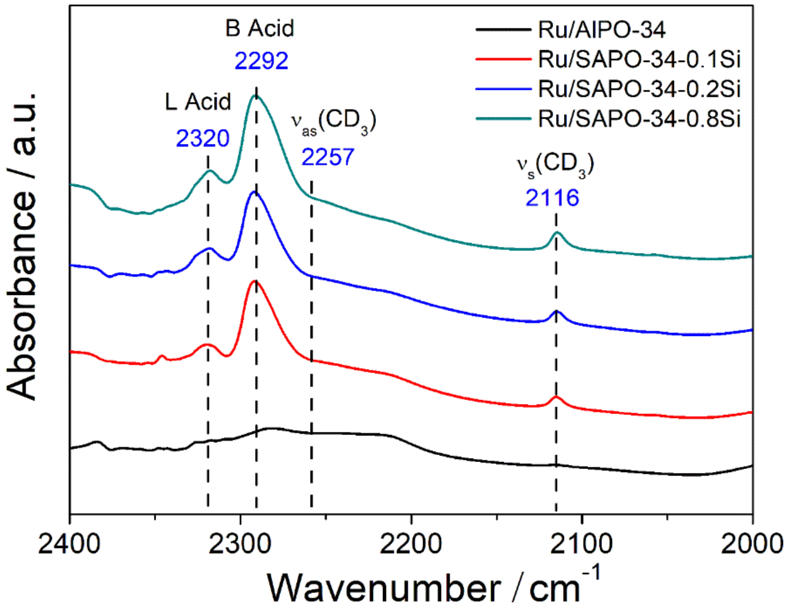

Figure S25. In-situ IR spectroscopy of the adsorbed deuterated CD_3CN of Ru/AlPO-34, Ru/SAPO-34-0.1Si, Ru/SAPO-34-0.2Si, and Ru/SAPO-34-0.8Si samples.

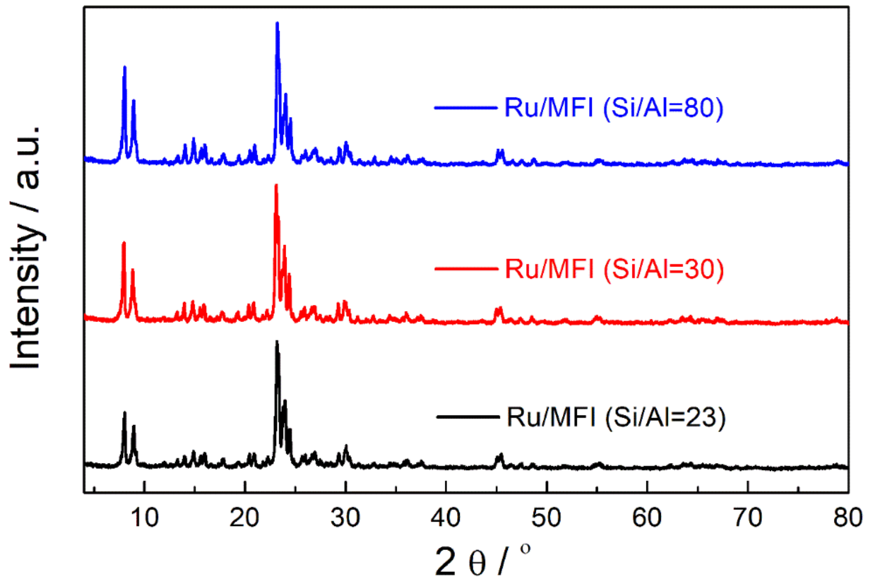

Figure S26. (a) XRD patterns of Ru/MFI catalysts with different ratio of Si/Al.

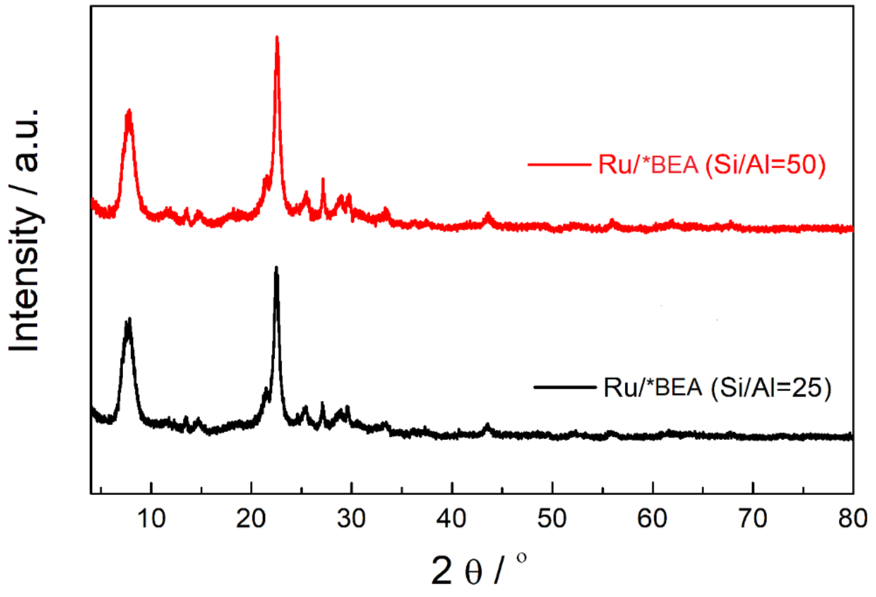

Figure S27. (a) XRD patterns of Ru/*BEA catalysts with different ratio of Si/Al.

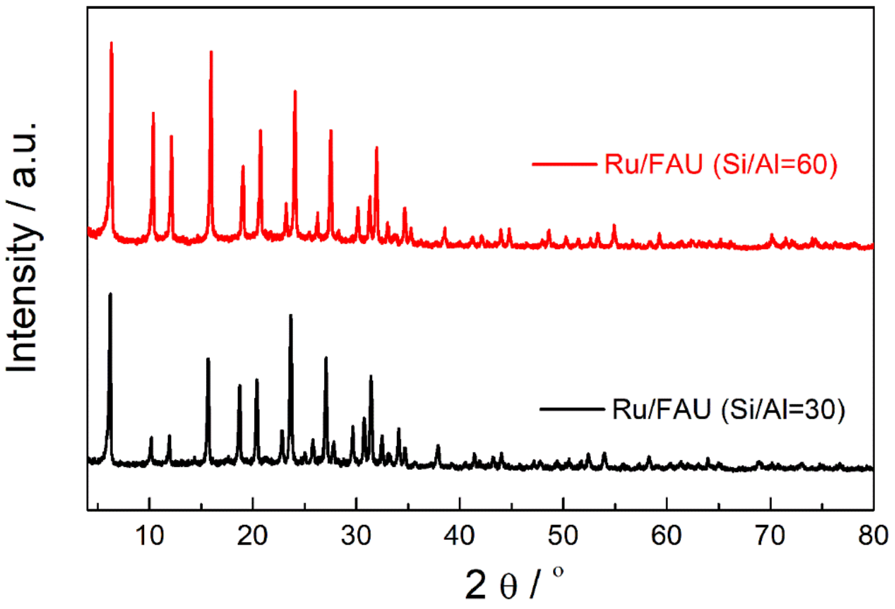

Figure S28. XRD patterns of Ru/FAU catalysts with different ratio of Si/Al.

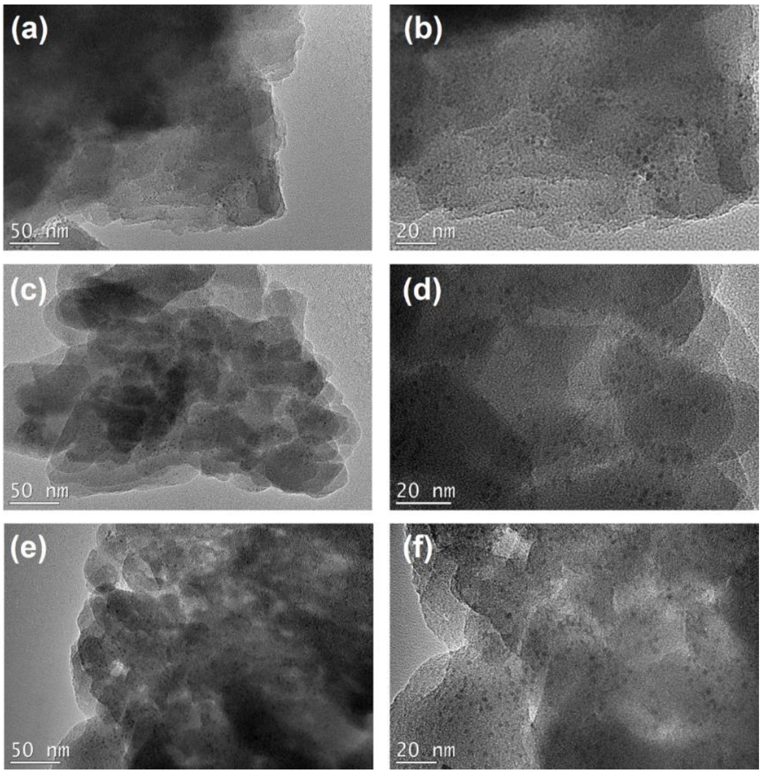

Figure S29. TEM images of (a, b) Ru/MFI (Si/Al=23), (c, d) Ru/MFI (Si/Al=30), and (e, f) Ru/MFI (Si/Al=80) catalysts.

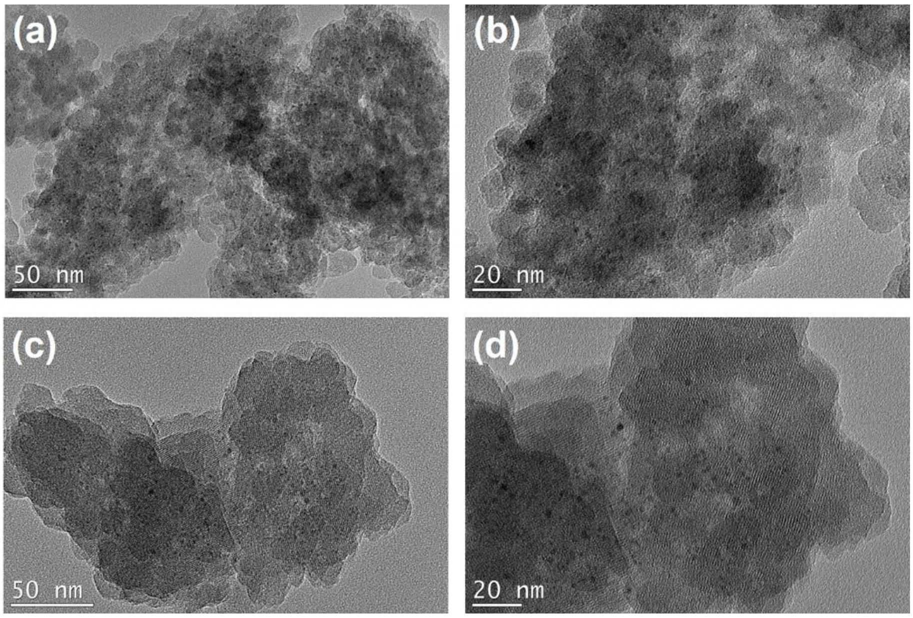

Figure S30. TEM images of (a, b) Ru/*BEA (Si/Al=25) and (c, d) Ru/*BEA (Si/Al=50).

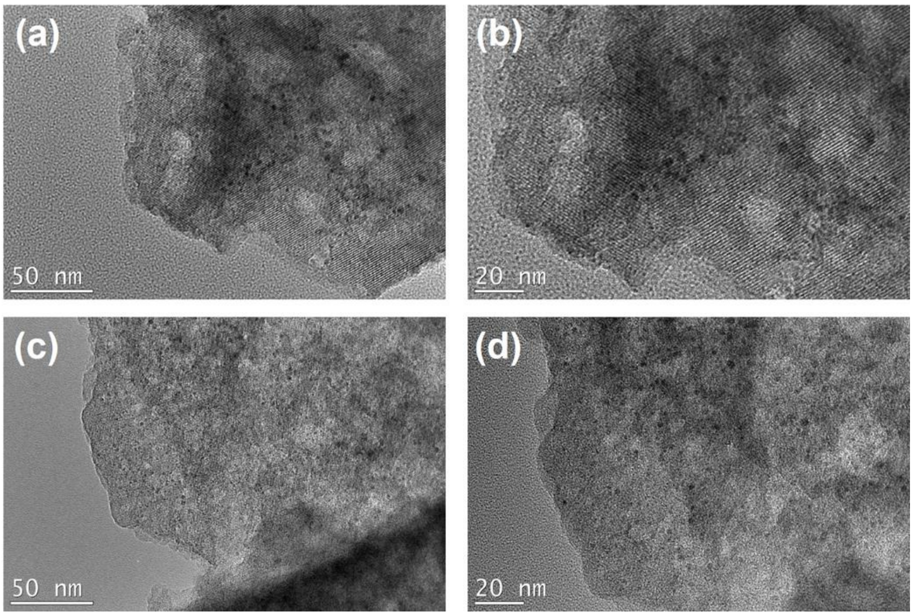

Figure S31. TEM images of (a, b) Ru/FAU (Si/Al=30) and (c, d) Ru/FAU (Si/Al=60).

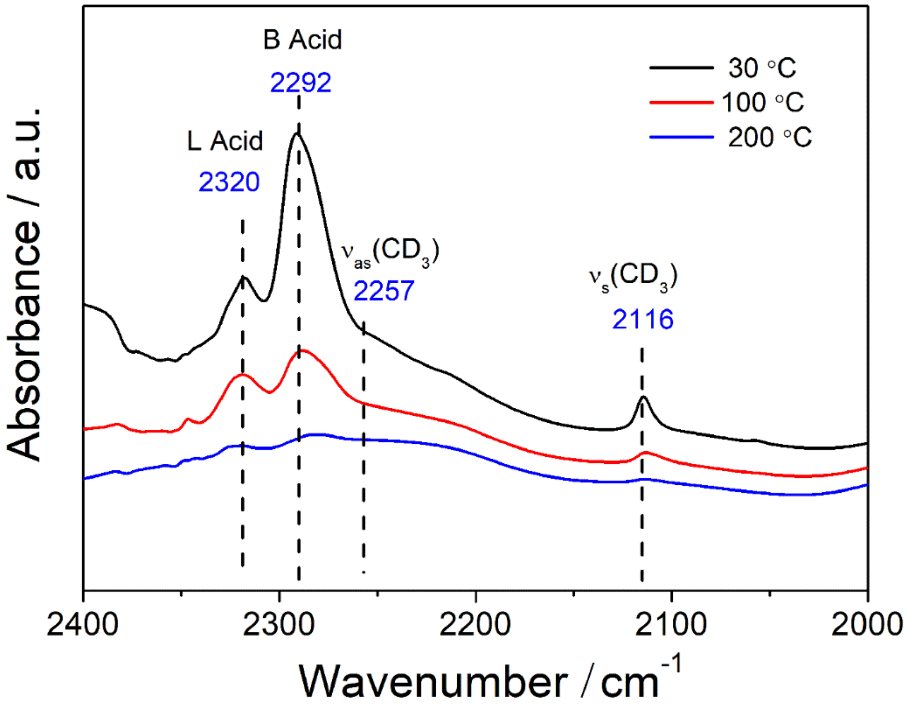

Figure S32. In-situ IR spectroscopy of the adsorbed deuterated CD_3CN of Ru/SAPO-34-0.8Si sample at different temperatures.

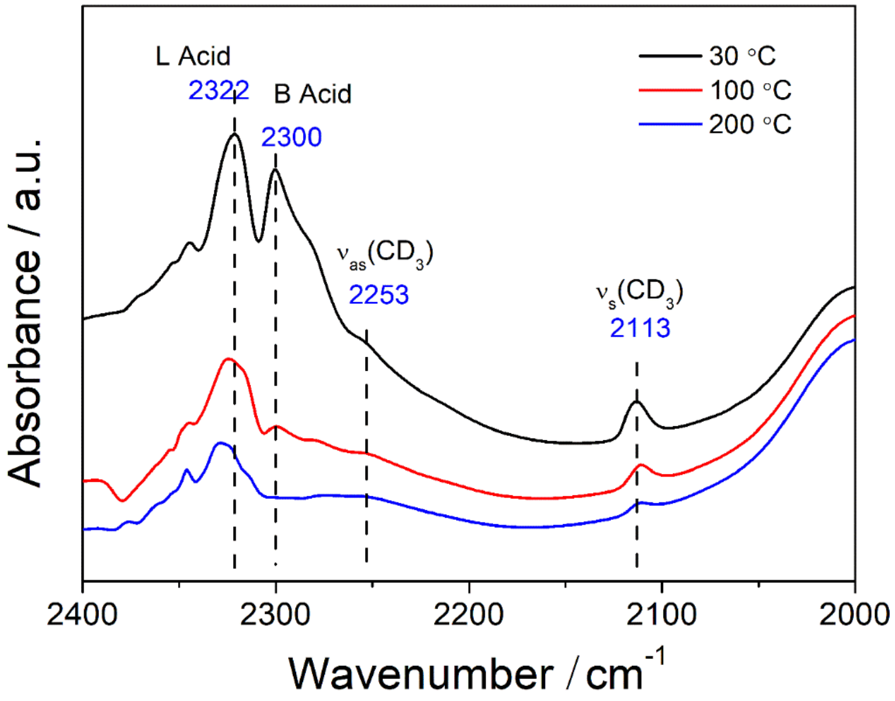

Figure S33. In-situ IR spectroscopy of the adsorbed deuterated CD_3CN of Ru/FAU (Si/Al = 30) sample at different temperatures.

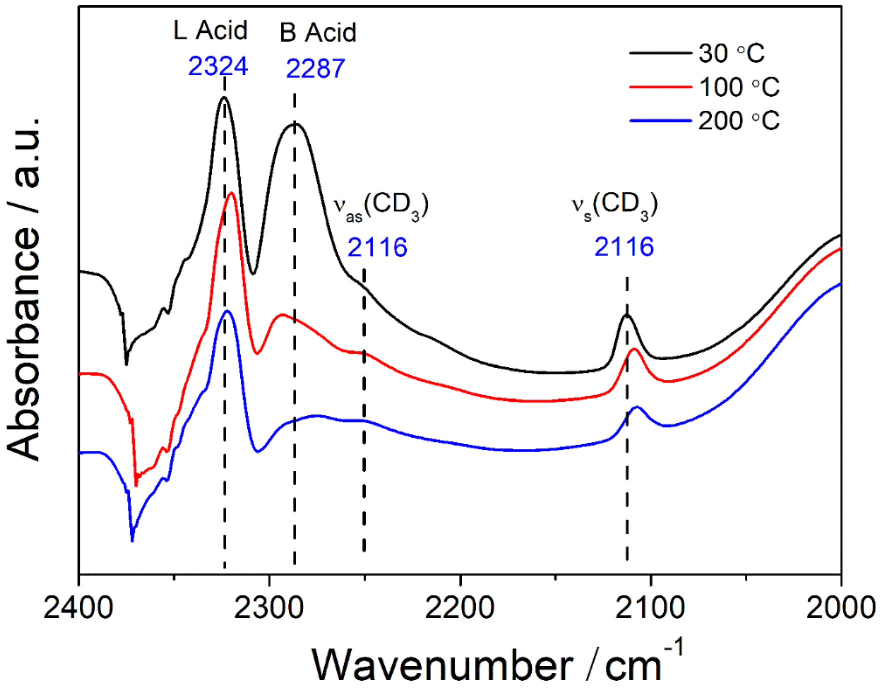

Figure S34. In-situ IR spectroscopy of the adsorbed deuterated CD_3CN of Ru/*BEA (Si/Al = 25) sample at different temperatures.

Table S1. Metal loading, porosity, TOF and acidity of the samples.

Sample	Metal loading [a] (wt %)	S_{total} [b] (m^2/g)	S_{micro} [c] (m^2/g)	S_{ext} [c] (m^2/g)	V_{micro} [c] (cm^3/g)	TOF [d] (min^{-1})	Total acid sites ($mmolg^{-1}$)[e]	B acid sites ($mmolg^{-1}$)[e]	L acid sites ($mmolg^{-1}$)[e]
AlPO-34	0	593	568	25	0.27	-	-	-	-
Ru/AlPO-34	0.43	517	497	20	0.24	118	Trace	Trace	Trace
SAPO-34-0.1Si	0	586	572	14	0.27	-	-	-	-
Ru/SAPO-34-0.1Si	0.44	511	492	19	0.24	205	0.42	0.40	0.02
SAPO-34-0.2Si	0	649	632	17	0.30	-	-	-	-
Ru/SAPO-34-0.2Si	0.44	537	516	21	0.25	310	0.57	0.49	0.08

[a] Analyzed by inductively coupled plasma atomic emission spectroscopy (ICP-AES). [b] S_{BET} (total surface area) calculated by applying the BET equation using the linear part ($0.05<P/P_0<0.30$) of the adsorption isotherm. [c] S_{micro} (micropore area) and S_{ext} (external surface area) calculated using the t-plot method. [d] TOF are values of H_2 generation for the AB hydrolysis at 25 °C. [e] Analyzed by in-situ FTIR spectra of surface species derived from CD_3CN-adsorption

Table S2. EXAFS parameters of Ru/AlPO-34, Ru/SAPO-34-0.1Si and Ru/SAPO-34-0.2Si samples.

	Shell	CN[a]	σ^2	R (Å)[b]
Ru/AlPO-34	Ru-O	3.8 ± 0.4	0.008± 0.002	2.01± 0.01
	Ru-Ru	1.8± 0.7	0.009± 0.004	2.69± 0.02
Ru/SAPO-34-0.1Si	Ru-O	3.5± 0.4	0.009± 0.002	2.01± 0.01
	Ru-Ru	2.0± 0.6	0.008± 0.003	2.68± 0.01
Ru/SAPO-34-0.2Si	Ru-O	3.0± 0.4	0.008± 0.002	2.01± 0.01
	Ru-Ru	2.1± 0.6	0.006± 0.002	2.68± 0.01

[a] CN, coordination number;

[b] R, distance between absorber and backscatter atoms.

Table S3. Comparisons of catalytic activities for the hydrolysis of AB catalyzed by previously reported heterogeneous catalysts with that synthesized in this work.

Catalysts	Temperature / K	$TOF_{Total}{}^{a}$ /min^{-1}	Reference
**Ru-based Catalysts**			
**Commercial Ru/C catalyst**	**298**	**46**	**This work**
**Ru/AlPO-34**	**298**	**118**	
**Ru/SAPO-34-0.1Si**	**298**	**205**	
**Ru/SAPO-34-0.2Si**	**298**	**310**	
**Ru/SAPO-34-0.4Si**	**298**	**356**	
**Ru/SAPO-34-0.6Si**	**298**	**415**	
**Ru/SAPO-34-0.8Si**	**298**	**490**	
**Ru/MFI (Si/Al =80)**	**298**	**302**	
**Ru/MFI (Si/Al =30)**	**298**	**497**	
**Ru/MFI (Si/Al =23)**	**298**	**575**	
**Ru/Beta (Si/Al =50)**	**298**	**501**	
**Ru/Beta (Si/Al =25)**	**298**	**615**	
**Ru/FAU (Si/Al =60)**[b]	**298**	**522**	
**Ru/FAU (Si/Al =30)**[b]	**298**	**627**	
LDO-Ru-FCC	298	1.8	S1
Ru NPs-H_3PO_4	298	6.67	S2
metastable RuNPs	298	21.8	S3
PVP-stabilized Ruthenium	298	47.7	S4
Ru@Al_2O_3	298	83.3	S5
MMT stabilized Ru	298	90.9	S6
Ru@MIL-101	298	123	S7
Ru@HAp	298	137	S8
Ru@PSSA-co-MA	298	187.6	S9
Ru/TiO_2	298	241	S10
RuNPs/MCNT	298	329	S11
Ru NPs@PCC-2	298	304.4	S12
$Ru_1Co_9/Ti_3C_2X_2$	298	187.5	S13
Ni-Ru alloy NPs	298	195	S14
Ru@Ni/C	298	250.1	S15
Ru@Ni/graphene	298	340	S16
RuRh@PVP	298	386	S17
**Other noble-metal-based Catalysts**			
Rh_1/VO_2	298	72	S18
Rh/CC3-R-homo	298	215.3	S19
Rh/P(triaz)	298	260	S20
Pt_{20}/CNT	298	416.5	S21
RhNi/graphene	298	420	S22
$Pt_{58}Ni_{33}Au_9$	298	496	S23

a. TOF_{Total} is the total turnover frequency when the conversion of AB reaches 100%.
b. The commercial **FAU** zeolites are dealuminated H-type zeolite Y.

Supplemental References

S1 H. Ma, C. Na, ACS Catal. **2015**, 5, 1726-1735.

S2 S. Caliskan, M. Zahmakiran, F. Durap, S. Ozkar, Dalton Trans. **2012**, 41, 4976-4984.

S3 E. K. Abo-Hamed, T. Pennycook, Y. Vaynzof, C. Toprakcioglu, A. Koutsioubas, O. A. Scherman, Small **2014**, 10, 3145-3152.

S4 H. Erdogan, O. Metin, S. Ozkar, Catal. Today **2011**, 170, 93-98.

S5 H. Can, O. Metin, Appl. Catal. B-environ **2012**, 125, 304-310.

S6 H.-B. Dai, X.-D. Kang, P. Wang, Int. J. Hydrogen Energy **2010**, 35, 10317-10323.

S7 N. Cao, T. Liu, J. Su, X. Wu, W. Luo, G. Cheng, New J. Chem. **2014**, 38, 4032-4035.

S8 S. Akbayrak, P. Erdek, S. Ozkar, Appl. Catal. B-environ **2013**, 142, 187-195.

S9 O. Metin, S. Sahin, S. Ozkar, Int. J. Hydrogen Energy **2009**, 34, 6304-6313.

S10 S. Akbayrak, S. Tanyildizi, I. Morkan, S. Ozkar, Int. J. Hydrogen Energy **2014**, 39, 9628-9637.

S11 S. Akbayrak, S. Ozkar, ACS Appl. Mater. Interfaces **2012**, 4, 6302-6310.

S12 Y. Fang, J. Li, T. Togo, F. Jin, Z. Xiao, L. Liu, H. Drake, X. Lian, H.-C. Zhou, Chem **2018**, 4, 555-563.

S13 X. Li, C. Zeng, G. Fan, Int. J. Hydrogen Energy **2015**, 40, 9217-9224.

S14 G. Chen, S. Desinan, R. Rosei, F. Rosei, D. Ma, Chem. Eur. J. **2012**, 18, 7925-7930.

S15 N. Cao, J. Su, X. Hong, W. Luo, G. Cheng, Chem. Asian J. **2014**, 9, 562-571.

S16 N. Cao, J. Su, W. Luo, G. Cheng, Int. J. Hydrogen Energy **2014**, 39, 426-435.

S17 M. Rakap, J. Alloys Compd. **2015**, 649, 1025-1030.

S18 L. Wang, H. Li, W. Zhang, X. Zhao, J. Qiu, A. Li, X. Zheng, Z. Hu, R. Si, J. Zeng, Angew. Chem. Int. Ed. **2017**, 56, 4712-4718.

S19 J.-K. Sun, W.-W. Zhan, T. Akita, Q. Xu, J. Am. Chem. Soc. **2015**, 137, 7063-7066.

S20 J.-K. Sun, Z. Kochovski, W.-Y. Zhang, H. Kirmse, Y. Lu, M. Antonietti, J. Yuan, J. Am. Chem. Soc. **2017**, 139, 8971-8976.

S21 J. Zhang, C. Chen, S. Chen, Q. Hu, Z. Gao, Y. Li, Y. Qin, Catal. Sci. Technol. **2017**, 7, 322-329.

S22 J. Shen, N. Cao, Y. Liu, M. He, K. Hu, W. Luo, G. Cheng, Catal. Commun. **2015**, 59, 14-20.

S23 J.-X. Kang, T.-W. Chen, D.-F. Zhang, L. Guo, Nano Energy **2016**, 23, 145-152.
